# Temporal trends and demographic disparities in mortality with co-mention of chronic liver disease and pneumonia in the United States, 1999–2024

**DOI:** 10.1186/s12876-026-05402-3

**Published:** 2026-10-01

**Authors:** Abdul Moeez Awais, Vishan Das, Muhammad Wassam, Nawal Jahangir Mahsud, Fatima Sohail, Muhammad Touseef, Mustafa Saad, Abdul Raffay Awais, Muhammad Hanif Khan

**Affiliations:** 1 Islamic International Medical College, Riphah International University, Rawalpindi, Pakistan; 2 Liaquat University of Medical & Health Sciences, Jamshoro, Sindh Pakistan; 3 AL Tibri Medical College, Isra University, Karachi, Sindh Pakistan; 4 Jinnah Sindh Medical University, Karachi, Pakistan; 5 University College of Medicine and Dentistry, The University of Lahore, Lahore, Punjab Pakistan; 6 Spinghar University, Kabul, Afghanistan

**Keywords:** Chronic liver disease, Pneumonia, Mortality trends, Demographic disparities, Age-adjusted mortality, Epidemiology, United States

## Abstract

**Background:**

Chronic liver disease is associated with immune dysfunction and increased susceptibility to pneumonia, yet national mortality trends involving both conditions remain poorly characterized. We examined temporal trends and demographic disparities in mortality with co-mention of chronic liver disease and pneumonia in the United States from 1999 to 2024.

**Methods:**

Using data from the Centers for Disease Control and Prevention’s Wide-Ranging Online Data for Epidemiologic Research database, we identified deaths with co-mention of chronic liver disease and pneumonia as multiple causes of death. Age-adjusted mortality rates per 100,000 population were calculated and stratified by sex, age, race and ethnicity, region, and urbanization. Joinpoint regression was used to estimate annual and average annual percent change in mortality rates. Sensitivity analyses excluded deaths with a co-listed U07.1 (COVID-19) code, restricted to the pre-pandemic period, and applied broader diagnostic code definitions.

**Results:**

From 1999 to 2024, 81,759 deaths had co-mention of chronic liver disease and pneumonia. The overall mortality rate was stable from 1999 to 2018, rose sharply to a peak in 2021, and declined thereafter but remained above pre-pandemic levels through 2024; this pattern was retained across all sensitivity analyses. Males accounted for most deaths and consistently had higher mortality rates than females, though females showed a significant overall increase in mortality while males did not. Non-Hispanic American Indian/Alaska Native individuals had the highest mortality rate throughout most of the study period, with a sharp increase from 2018 to 2021 followed by a marked decline. The South accounted for the most deaths, while the West had the highest mortality rate by 2024. Non-metropolitan areas showed a greater increase in mortality than metropolitan areas. Most deaths occurred in inpatient medical facilities.

**Conclusions:**

Mortality with co-mention of chronic liver disease and pneumonia rose sharply during the COVID-19 pandemic and remained persistently elevated through 2024; this pattern was similar in sensitivity analyses excluding deaths with a co-listed U07.1 code and using alternative case definitions. Substantial and sustained disparities were observed, particularly among non-Hispanic American Indian/Alaska Native individuals and non-metropolitan residents. These findings reflect co-occurrence on death certificates rather than a confirmed causal relationship, and highlight priority populations for continued surveillance and targeted public health efforts.

**Clinical Trial Number:**

Not applicable.

**Graphical abstract:**

**Figure:**
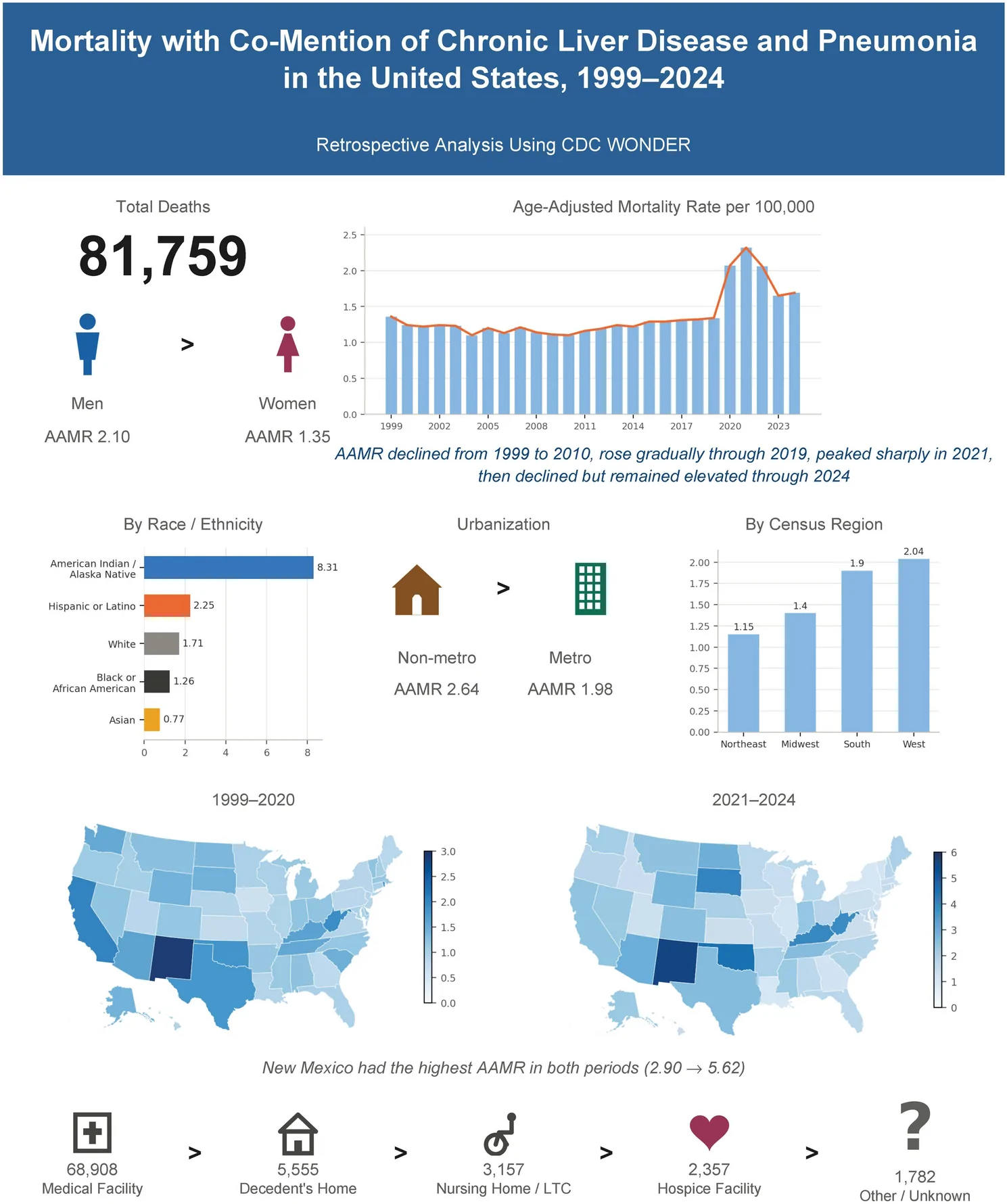

**Supplementary Information:**

The online version contains supplementary material available at https://doi.org/10.1186/s12876-026-05402-3.

## Introduction

Pneumonia remains a leading cause of morbidity and mortality in the United States, and this burden is amplified among patients with concurrent chronic conditions [1]. As the population ages and multimorbidity becomes more common, understanding how such comorbidities shape pneumonia mortality is increasingly important [2].

Chronic liver disease (CLD), including alcoholic liver disease, non-viral chronic hepatitis, and hepatic fibrosis/cirrhosis, is itself a leading cause of death nationally, ranking 9th as of 2023, driven in part by rising rates of metabolic dysfunction-associated steatotic liver disease (MASLD/NAFLD) [3, 4]. Patients with CLD experience worse outcomes across a range of hospital presentations, including pneumonia [5]. This vulnerability reflects several converging mechanisms: cirrhosis-associated immune dysfunction impairs pathogen clearance [6]; hepatic encephalopathy increases aspiration risk by impairing consciousness and airway-protective reflexes [7]; ascites and hepatic hydrothorax mechanically restrict lung expansion [8]; and portosystemic shunting allows gut-derived bacteria to bypass hepatic clearance, raising infection risk [9]. Together, these mechanisms provide a plausible biological basis for elevated pneumonia mortality in this population.

While this relationship is established at the individual patient level, whether it has translated into a shifting national mortality burden over time is unclear. Trend analysis — rather than a single cross-sectional association — is needed to anticipate healthcare demand, identify inflection points tied to events such as the COVID-19 pandemic, and target populations experiencing disproportionate increases. Prior studies have not well characterized these national trends across demographic groups. We therefore used the CDC Wide-Ranging Online Data for Epidemiologic Research (WONDER) database to examine temporal trends in mortality with co-mention of CLD and pneumonia in the United States from 1999 to 2024, evaluating differences by age, sex, race, region, and urbanization to identify populations at greatest risk.

## Methods

### Study design and population

Data for this analysis were derived from the Multiple Cause-of-Death Public Use files accessible via the Centers for Disease Control and Prevention (CDC) Wide-Ranging Online Data for Epidemiologic Research (WONDER) database [10]. The study population comprised U.S. residents aged 25 to 85 + years who died between 1999 and 2024 and had both chronic liver disease and pneumonia co-mention among the multiple causes of death on their death certificates. Chronic liver disease was defined by the presence of ICD-10 codes K70 (alcoholic liver disease), K73 (chronic hepatitis, not elsewhere classified), and K74 (fibrosis and cirrhosis of the liver), while pneumonia was captured using codes J12–J18, covering viral, bacterial, and unspecified pneumonia etiologies [11]. Chronic liver disease was restricted to ICD-10 codes K70, K73, and K74 to capture alcoholic liver disease, chronic hepatitis, and hepatic fibrosis/cirrhosis, the most commonly reported forms of chronic liver disease in mortality surveillance. The narrower definition was selected as the primary case definition because K70, K73, and K74 represent more clinically specific markers of established, structural chronic liver disease, particularly alcoholic liver disease and cirrhosis. In contrast, B18 (chronic viral hepatitis) can reflect earlier-stage or asymptomatic infection without necessarily indicating advanced liver damage, and K76 is a heterogeneous residual category; both were reserved for the broader sensitivity analysis rather than the primary definition to avoid diluting specificity with conditions of variable severity. This definition does not capture other clinically important forms of chronic liver disease, including K71 (toxic liver disease), K72 (hepatic failure, not elsewhere classified), K75 (other inflammatory liver diseases), K76 (other diseases of liver, including MASLD/NAFLD), and B18 (chronic viral hepatitis B and C); their exclusion may result in under-ascertainment of the broader chronic liver disease spectrum, particularly MASLD/NAFLD and viral hepatitis, both of which are increasingly prevalent causes of chronic liver disease [4]. Pneumonia was defined using the broad J12–J18 code range, encompassing viral, bacterial, and unspecified pneumonia, in order to maximize sensitivity for pneumonia-related mortality; this breadth may reduce specificity relative to narrower pneumonia definitions.

Death certificates were eligible if chronic liver disease and pneumonia codes were both listed as multiple (contributing) causes of death, regardless of which condition was designated as the underlying cause. Data were obtained from the CDC WONDER Multiple Cause of Death (final, non-provisional) database, querying the Multiple Cause of Death Files for 1999–2020 (bridged-race) and 2018–2024 (single-race). Because these two extracts overlapped for years 2018–2020, records for these years were drawn from the 1999–2020 file only, and the 2018–2024 file was used exclusively for 2021–2024 data, to construct a non-duplicated analytic dataset spanning 1999–2024. Mortality data for all study years, including 2024, were obtained from CDC WONDER’s final (non-provisional) Multiple Cause of Death files rather than the Provisional Mortality Statistics database, and thus represent finalized death certificate data throughout the full 1999–2024 study period. As a sensitivity analysis, trends were additionally examined using broader diagnostic code ranges (K70–K76 and B18 for liver disease; J09–J18 for pneumonia) to assess the influence of the narrower primary case definition on observed trends. This coding approach has been applied in prior epidemiologic studies analyzing mortality trends in comorbid chronic disease populations. This study was designed as a retrospective, observational analysis, conducted in accordance with the STROBE (Strengthening the Reporting of Observational Studies in Epidemiology) guidelines to ensure methodological transparency and consistency in reporting [12].

### Data abstraction

This study’s primary data source was the CDC WONDER Database, which provides online access to publicly available public health data and analytical tools, supporting evidence-based assessment of public health policies and demographic trends. Data on population size, year, location of death, demographics, metropolitan/non-metropolitan classification, census region, and state were abstracted. Demographic data consisted of sex, age, and race. The sex variable included male and female, as specified on the death certificates. Age was categorized into the following groups: 25–34, 35–44, 45–54, 55–64, 65–74, 75–84, and 85 + years. Race was categorized into non-Hispanic White, non-Hispanic Black or African American, non-Hispanic American Indian or Alaska Native (AI/AN), Hispanic or Latino, and non-Hispanic Asian or Pacific Islander. Race was classified using CDC WONDER’s bridged-race categories for 1999–2020 and single-race categories for 2021–2024. The NH Asian and Native Hawaiian/Other Pacific Islander single-race categories (2021–2024) were combined into a single ' NH Asian or Pacific Islander’ category to maintain consistency with the bridged-race classification used in earlier years. Because CDC WONDER suppressed year-specific Native Hawaiian/Other Pacific Islander counts for most years due to small cell sizes, the suppressed total (*n* = 42 across 2018–2023) was prorated across affected years to estimate this subgroup’s contribution for 2021–2024 (see Table 1 for details). For years 2018–2020, which were present in both WONDER extracts, data were drawn from the 1999–2020 bridged-race file only to avoid duplication. Hispanic origin data for 2018–2020 were similarly drawn from the 1999–2020 file only, with the 2018–2024 file used for 2021–2024. Regions were categorized according to the U.S. Census Bureau’s directions, which classified them as Northeast, Midwest, South and West. Urbanization was categorized using the 2013 NCHS Urban-Rural Classification Scheme, which groups U.S. counties into six levels from large central metro areas to noncore non-metropolitan counties based on population size and geographical data that were abstracted into census regions and states [13]. Four U.S. census regions were used for analysis: Northeast, Midwest, South, and West, each including specific states as defined by the Census Bureau. It is worth noting that CDC WONDER does not provide urbanization classification data beyond 2020, so analyses stratified by urbanization level were limited to the 1999–2020 period. Place of death was categorized as Medical Facility (further subdivided into inpatient, outpatient/emergency room, and dead-on-arrival), Decedent’s Home, Hospice Facility, Nursing Home/Long-Term Care, Other, and Unknown.

**Table 1:** Absolute number of deaths with co-mention of CLD and pneumonia and percent of total deaths stratified by sex, race/ethnicity, age group, census region, urbanization, and place of death in the United States, 1999–2024

Variables	Deaths	% of Total Deaths
Sex		
Overall	81,759	100%
Male	50,682	61.98%
Female	31,077	38.01%
Race/Ethnicity		
NH White	56,039	68.54%
NH Black or African American	7,527	9.20%
NH American Indian or Alaska Native	2,403	2.93%
NH Asian or Pacific Islander	1,825	2.23%
NH More than one race	218	0.26%
Hispanic or Latino	13,466	16.47%
Age Groups		
Young adults (25–34 years)	1,434	1.75%
Adults (35–44 years)	6,091	7.44%
Middle-aged adults (45–54 years)	16,899	20.66%
Older adults (55–64 years)	24,555	30.03%
Young-old adults (65–74 years)	18,810	23.00%
Middle-old adults (75–84 years)	10,543	12.89%
Oldest-old adults (85 + years)	3,427	4.19%
Census Region		
Northeast	11,143	13.62%
Midwest	14,802	18.10%
South	31,588	38.63%
West	24,226	29.63%
Urbanization		
Metropolitan	50,637	82.68%
Non-metropolitan	10,612	17.32%
Place of Death		
Medical Facility – Inpatient	66,833	81.74%
Medical Facility – Outpatient or ER	2,075	2.53%
Decedent’s Home	5,555	6.79%
Hospice Facility	2,357	2.88%
Nursing Home / Long-term Care	3,157	3.86%
Other / Unknown	1,782	2.18%

NH: Non-Hispanic. Overlapping years across WONDER extracts were deduplicated (see Methods). Race/ethnicity categories reflect ICD coding changes over the study period; the NH Asian or Pacific Islander category combines Asian and Native Hawaiian or Other Pacific Islander subgroups. Race/ethnicity categories may not sum to the overall total due to a small number of deaths with race not stated. For 2021–2023, CDC WONDER suppressed year-specific Native Hawaiian/Other Pacific Islander counts due to small cell sizes; the NH Asian or Pacific Islander total for these years therefore includes a prorated estimate of the suppressed deaths (based on *n* = 42 suppressed across 2018–2023) and should be interpreted as approximate. Place of death “Other/Unknown” combines Dead on Arrival, Status Unknown, and Other categories; place of death categories may not sum to the overall total due to suppression of small cell counts. Urbanization percentages are calculated using the 1999–2020 total (*N* = 61,249) as the denominator, consistent with data availability for that period

### Statistical analysis

We assessed national mortality patterns for co-mention of chronic liver disease and pneumonia by calculating crude and age-adjusted mortality rates (AAMRs) per 100,000 population from 1999 to 2024 [14]. Mortality rates were stratified by calendar year, sex, race, state, and metropolitan/non-metropolitan classification, with 95% confidence intervals (CIs) reported. Crude rates were obtained by dividing the number of deaths attributed to chronic liver disease-associated pneumonia by the corresponding U.S. population for each year. Age-adjusted rates were calculated using the direct method, standardized to the 2000 U.S. standard population. Age-specific mortality rates are presented as crude rates, as age-adjustment is not applicable within a single age stratum. The 25–34-year age group was excluded from Joinpoint trend analysis in its entirety, as CDC WONDER flagged annual rates for 2004–2006 as unreliable due to small case counts; given the consistently low annual death counts in this age group across the study period, the reliability of a segmented trend model was judged insufficient. Crude death counts for this group are reported in Table 1. More broadly, data points with CDC WONDER-flagged unreliable or suppressed rates were excluded from Joinpoint modeling for the corresponding stratum.

Trends in AAMRs over the study period were assessed using the Joinpoint Regression Program (Version 5.4.0, National Cancer Institute) [15], allowing a maximum of 4 joinpoints, with rates log-transformed prior to modeling. Model errors were assumed to be independent and homoscedastic (constant variance), the default error structure in Joinpoint; no additional weighting beyond this modeled variance structure was applied. This program fits log-linear models to detect statistically significant changes in mortality trends over time. Annual percent change (APC) with 95% confidence intervals was calculated to measure the direction and magnitude of trend shifts, with APCs classified as increasing or decreasing based on their deviation from zero. Confidence intervals were calculated using the empirical quantile method. The Monte Carlo Permutation Test, based on 4,499 permutations (the Joinpoint default), was used as the primary criterion for model selection to determine the final number of joinpoints. A p-value of less than 0.05 was considered statistically significant. AAPC and 95% CIs for all subgroups are reported in Table 2.

**Table 2:** Annual percent change (APC) in age-adjusted mortality rates per 100,000 for deaths with co-mention of CLD and pneumonia, by joinpoint-defined year interval, overall and by sex, region, metropolitan/non-metropolitan status, race/ethnicity, and age group, United States, 1999–2024

Subgroup	Year Interval	APC (95% CI)	*p*-Value
Overall			
	1999–2018	0.33 (-0.26 to 0.91)	0.258
	2018–2021	23.25* (5.70 to 43.71)	0.010
	2021–2024	-11.78* (-18.91 to -4.02)	0.006
Female			
	1999–2009	-0.49 (-1.79 to 0.83)	0.439
	2009–2018	3.52* (1.83 to 5.24)	< 0.001
	2018–2021	20.79* (8.54 to 34.41)	0.002
	2021–2024	-10.76* (-15.19 to -6.09)	< 0.001
Male			
	1999–2018	-0.61 (-1.22 to 0.00)	0.051
	2018–2021	22.91* (3.22 to 46.36)	0.023
	2021–2024	-12.15* (-18.71 to -5.07)	0.003
Northeast			
	1999–2008	-3.31* (-5.23 to -1.35)	0.003
	2008–2018	1.81 (-0.21 to 3.86)	0.075
	2018–2021	14.92 (-6.24 to 40.85)	0.166
	2021–2024	-10.43* (-17.73 to -2.48)	0.015
Midwest			
	1999–2009	-1.32* (-2.61 to -0.02)	0.046
	2009–2018	2.06* (0.33 to 3.82)	0.023
	2018–2021	22.44* (7.86 to 39.00)	0.004
	2021–2024	-12.25* (-17.17 to -7.04)	< 0.001
South			
	1999–2010	-1.14 (-2.39 to 0.13)	0.076
	2010–2018	2.98* (0.59 to 5.44)	0.018
	2018–2021	20.91* (5.61 to 38.43)	0.009
	2021–2024	-10.06* (-15.14 to -4.67)	0.002
West			
	1999–2018	-0.53* (-1.01 to -0.06)	0.030
	2018–2021	23.27* (7.86 to 40.88)	0.004
	2021–2024	-13.60* (-18.88 to -7.97)	< 0.001
Metropolitan			
	1999–2018	-0.16 (-0.69 to 0.36)	0.522
	2018–2020	24.74* (10.65 to 40.62)	0.001
Non-Metropolitan			
	1999–2010	-1.29* (-2.45 to -0.12)	0.033
	2010–2018	4.80* (2.60 to 7.05)	< 0.001
	2018–2020	24.82* (9.96 to 41.68)	0.002
NH American Indian/Alaska Native			
	1999–2018	2.78* (1.41 to 4.17)	< 0.001
	2018–2021	42.72* (5.88 to 92.39)	0.022
	2021–2024	-23.08* (-32.91 to -11.82)	< 0.001
NH Asian or Pacific Islander			
	1999–2011	-4.68* (-8.06 to -1.18)	0.012
	2011–2024	4.19* (1.76 to 6.69)	0.002
NH Black or African American			
	1999–2010	-4.35* (-6.15 to -2.51)	< 0.001
	2010–2018	2.00 (-1.65 to 5.78)	0.264
	2018–2021	17.38 (-8.46 to 50.52)	0.190
	2021–2024	-10.64* (-19.25 to -1.12)	0.032
NH White			
	1999–2009	-1.15* (-2.20 to -0.09)	0.036
	2009–2018	1.93* (0.41 to 3.47)	0.016
	2018–2021	19.93* (8.31 to 32.80)	0.002
	2021–2024	-8.74* (-13.06 to -4.21)	0.001
Hispanic or Latino			
	1999–2018	-0.54 (-1.40 to 0.33)	0.207
	2018–2021	27.08* (3.54 to 55.96)	0.024
	2021–2024	-20.99* (-28.12 to -13.15)	< 0.001
35–44 years			
	1999–2009	-4.30* (-5.93 to -2.64)	< 0.001
	2009–2018	2.73* (0.17 to 5.34)	0.038
	2018–2021	27.00* (2.21 to 57.80)	0.033
	2021–2024	-10.33* (-18.12 to -1.80)	0.022
45–54 years			
	1999–2018	-0.43 (-0.89 to 0.03)	0.065
	2018–2021	19.00* (3.06 to 37.39)	0.020
	2021–2024	-14.21* (-19.81 to -8.22)	< 0.001
55–64 years			
	1999–2018	2.24* (1.58 to 2.91)	< 0.001
	2018–2021	16.80 (-0.12 to 36.58)	0.052
	2021–2024	-14.53* (-20.69 to -7.89)	< 0.001
65–74 years			
	1999–2009	-2.88* (-4.66 to -1.06)	0.004
	2009–2018	3.09* (0.76 to 5.48)	0.013
	2018–2021	23.43* (6.07 to 43.64)	0.010
	2021–2024	-11.63* (-17.38 to -5.47)	0.001
75–84 years			
	1999–2010	-4.00* (-5.48 to -2.50)	< 0.001
	2010–2018	3.11 (-0.11 to 6.43)	0.057
	2018–2021	19.13 (-1.49 to 44.06)	0.068
	2021–2024	-6.45 (-13.35 to 0.99)	0.083
85 + years			
	1999–2018	-2.05* (-3.07 to -1.01)	0.001
	2018–2021	28.08 (-4.38 to 71.58)	0.092
	2021–2024	-1.84 (-13.09 to 10.86)	0.752

Given concern that the 2018–2021 rise in mortality may partly reflect COVID-19-associated pneumonia, a sensitivity analysis was conducted excluding death certificates with a co-listed ICD-10 code U07.1 (COVID-19), and Joinpoint trend analysis was repeated on this restricted dataset to assess whether observed trends were attributable to pandemic-related coding.

## Results

### Overall trend

**Fig. 1:**
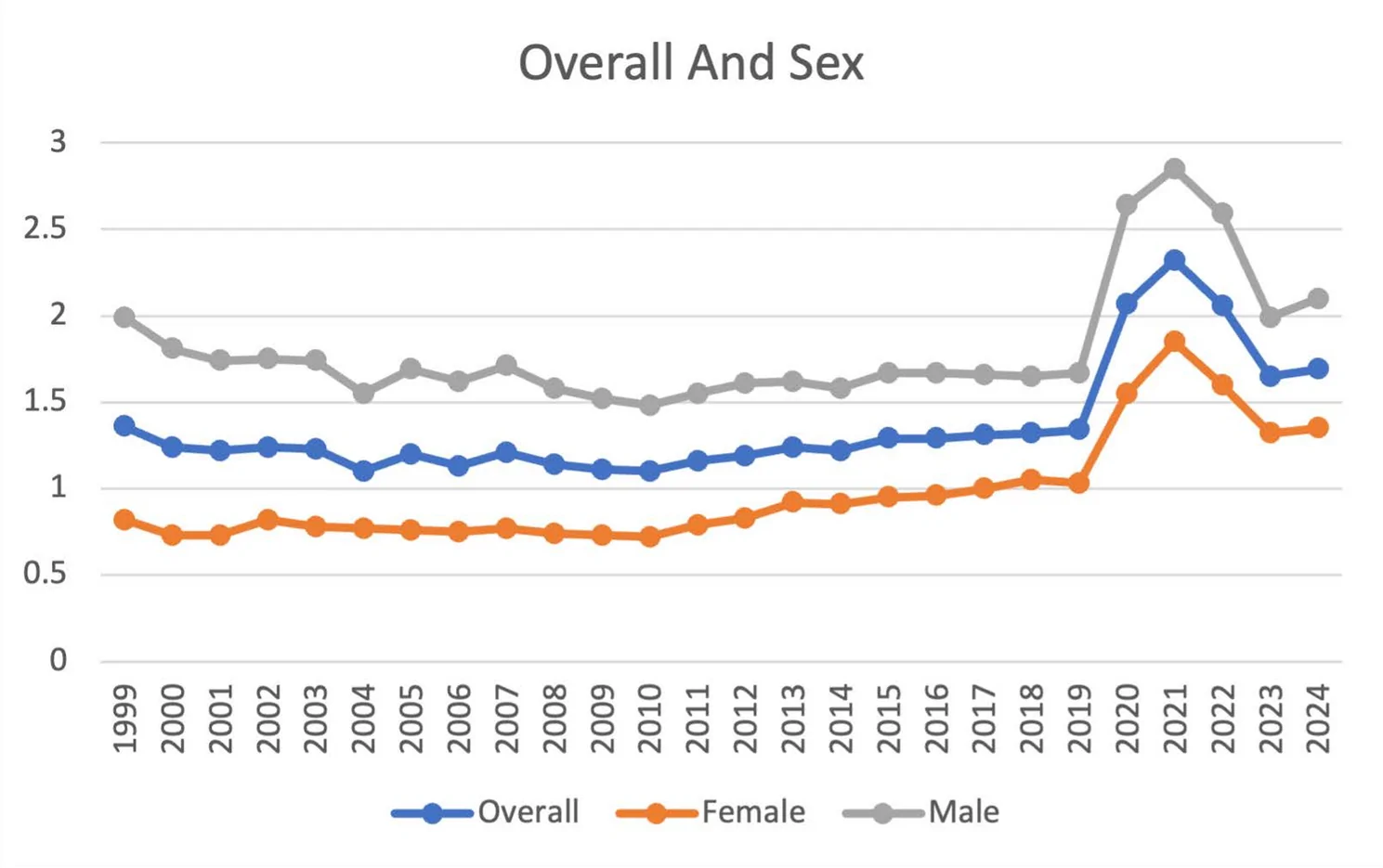
Overall and sex-stratified age-adjusted mortality rates (per 100,000) for deaths with co-mention of chronic liver disease and pneumonia among U.S. adults aged ≥ 25 years, 1999–2024

From 1999 to 2024, 81,759 deaths had co-mention of the selected CLD and pneumonia codes (Table S1). The overall AAMR was 1.36 (95% CI, 1.31–1.42) in 1999 and 1.32 (95% CI, 1.28–1.37) in 2018, corresponding to a nonsignificant APC of 0.33% (95% CI, − 0.26% to 0.91%). The AAMR increased to 2.32 (95% CI, 2.26–2.38) in 2021 (APC, 23.25%; 95% CI, 5.70%–43.71%) and then declined to 1.69 (95% CI, 1.64–1.74) in 2024 (APC, − 11.78%; 95% CI, − 18.91% to − 4.02%). The full-period AAPC was 1.26% (95% CI, − 0.75% to 3.31%; *p* = 0.221) (Fig. 1; Tables S2–S3).

### Stratified by pre-pandemic (1999–2019) and pandemic periods (2020–2022)

Using a calendar-period comparison, the mean of the annual AAMRs during 2020–2022 was 2.15 per 100,000, which was 1.76 times the mean pre-pandemic AAMR of 1.22 during 1999–2019 (rate ratio, 1.76). The AAMR increased by 54.5% from 1.34 in 2019 to 2.07 in 2020, increased by a further 12.1% to 2.32 in 2021, and then decreased by 11.2% to 2.06 in 2022 (Fig. 1; Table S2).

In the pre-pandemic-only Joinpoint analysis (1999–2019), the AAMR declined during 1999–2009 (APC, -1.36%; 95% CI, -2.22% to -0.49%; *p* = 0.005) and increased during 2009–2019 (APC, 1.98%; 95% CI, 1.26%-2.71%; *p* < 0.001). The overall pre-pandemic AAPC was 0.30% (95% CI, -0.22% to 0.82%; *p* = 0.263) and was not statistically significant (Table S11).

### COVID-19 underlying-cause and case-definition sensitivity analyses

During 2020–2024, 25,893 deaths met the primary co-mention definition. Of these, 5,222 (20.17%) were deaths in which COVID-19 (U07.1) was recorded as the underlying cause and the selected CLD and pneumonia codes were both co-mentioned among the multiple causes of death. No deaths met this U07.1-underlying definition in 2019. The annual proportions of all primary-definition deaths were 25.67% in 2020 (1,382/5,383), 34.45% in 2021 (2,096/6,085), 23.59% in 2022 (1,292/5,478), 6.19% in 2023 (268/4,331), and 3.99% in 2024 (184/4,616). The AAMRs for this U07.1-underlying subset were 0.51, 0.77, 0.47, 0.11, and 0.06 per 100,000, respectively (Table S11).

As a separate sensitivity analysis, death certificates with a co-listed U07.1 code (i.e., COVID-19 recorded anywhere among the multiple causes of death, regardless of whether it was the underlying cause) were excluded, and Joinpoint trend analysis was repeated on the resulting restricted dataset spanning the full 1999–2024 period. After this exclusion, the overall AAMR declined during 1999–2010 (APC, − 1.33%; 95% CI, − 2.21% to − 0.43%; *p* = 0.006) and increased during 2010–2024 (APC, 2.78%; 95% CI, 2.16%–3.39%; *p* < 0.001); the AAPC was 0.95% (95% CI, 0.46%–1.44%; *p* < 0.001). The 2018–2021 increase and 2021–2024 decline were also retained when CLD was restricted to K74 (APCs, 22.51% and − 12.50%), pneumonia was broadened to J09-J18 (19.37% and − 9.87%), or CLD was broadened to K70-K76 plus B18 (25.82% and − 14.31%) (Table S11).

### Sex

Males accounted for 50,682 deaths (61.99%) and females for 31,077 (38.01%). Male AAMRs exceeded female AAMRs every year. Both groups increased sharply during 2018–2021 and declined during 2021–2024. Across 1999–2024, the AAPC was significant among females (1.97%; 95% CI, 0.46%–3.51%; *p* = 0.011) but not males (0.46%; 95% CI, − 1.71% to 2.67%; *p* = 0.682) (Fig. 1; Tables S1–S3).

### Age

**Fig. 2:**
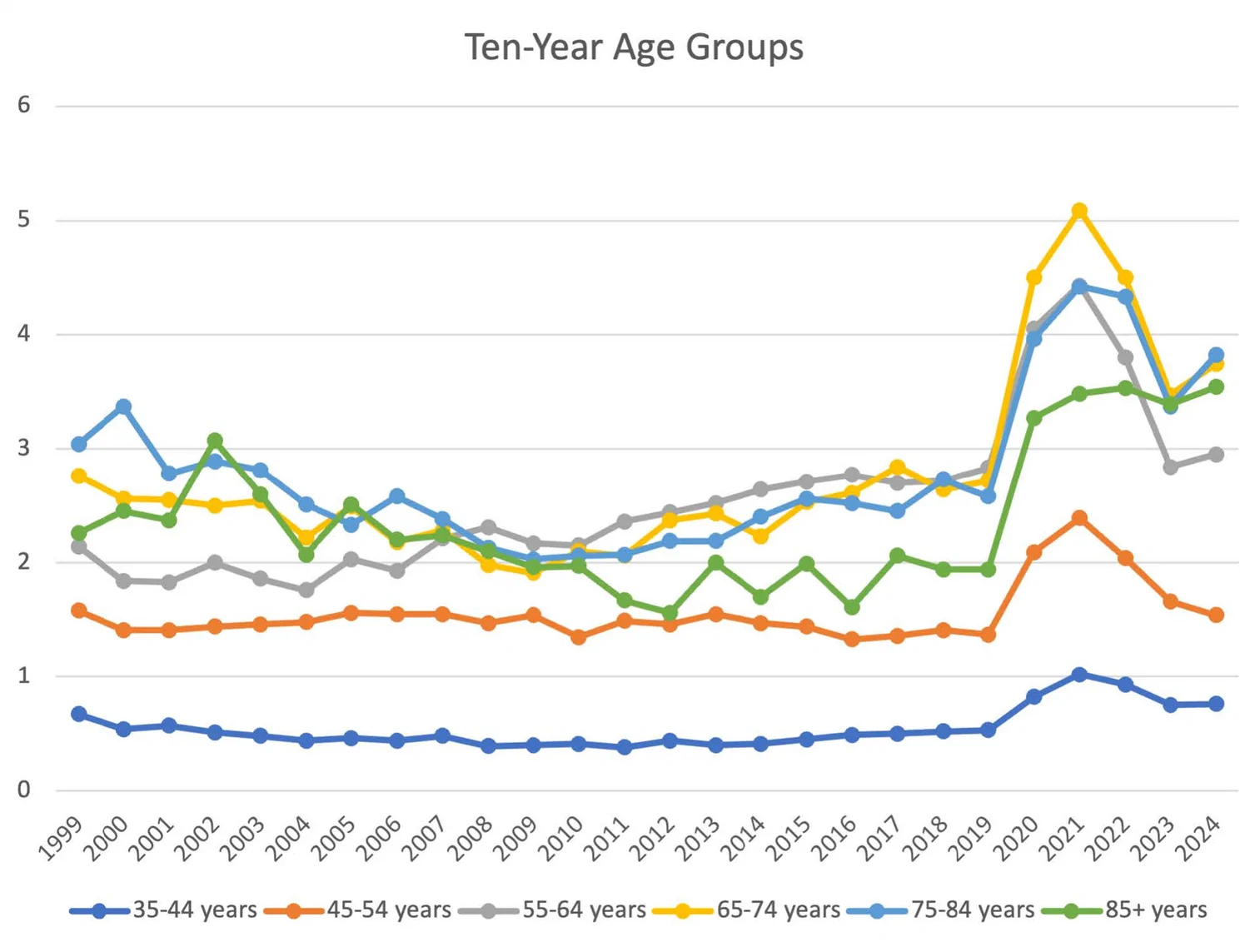
Age-stratified crude mortality rates (per 100,000) for deaths with co-mention of chronic liver disease and pneumonia among U.S. adults aged ≥ 25 years, 1999–2024

The largest shares of deaths occurred at ages 55–64 years (24,555; 30.03%) and 65–74 years (18,810; 23.01%) (Table S1). Annual crude rates generally rose with age from the 35–44-year group through the 65–74 and 75–84-year groups; however, the 85 + group’s rate was consistently lower than the 65–74 and 75–84-year groups throughout the study period, and the 35–44-year group consistently had the lowest modeled rates (Fig. 2; Table S4). For every modeled age group, rates rose during 2018–2021 and subsequently declined or stabilized. The 25–34-year group accounted for 1,434 deaths (1.75%); it was not included in Joinpoint regression because its 2004–2006 annual rates were flagged as unreliable.

### Race and ethnicity

**Fig. 3:**
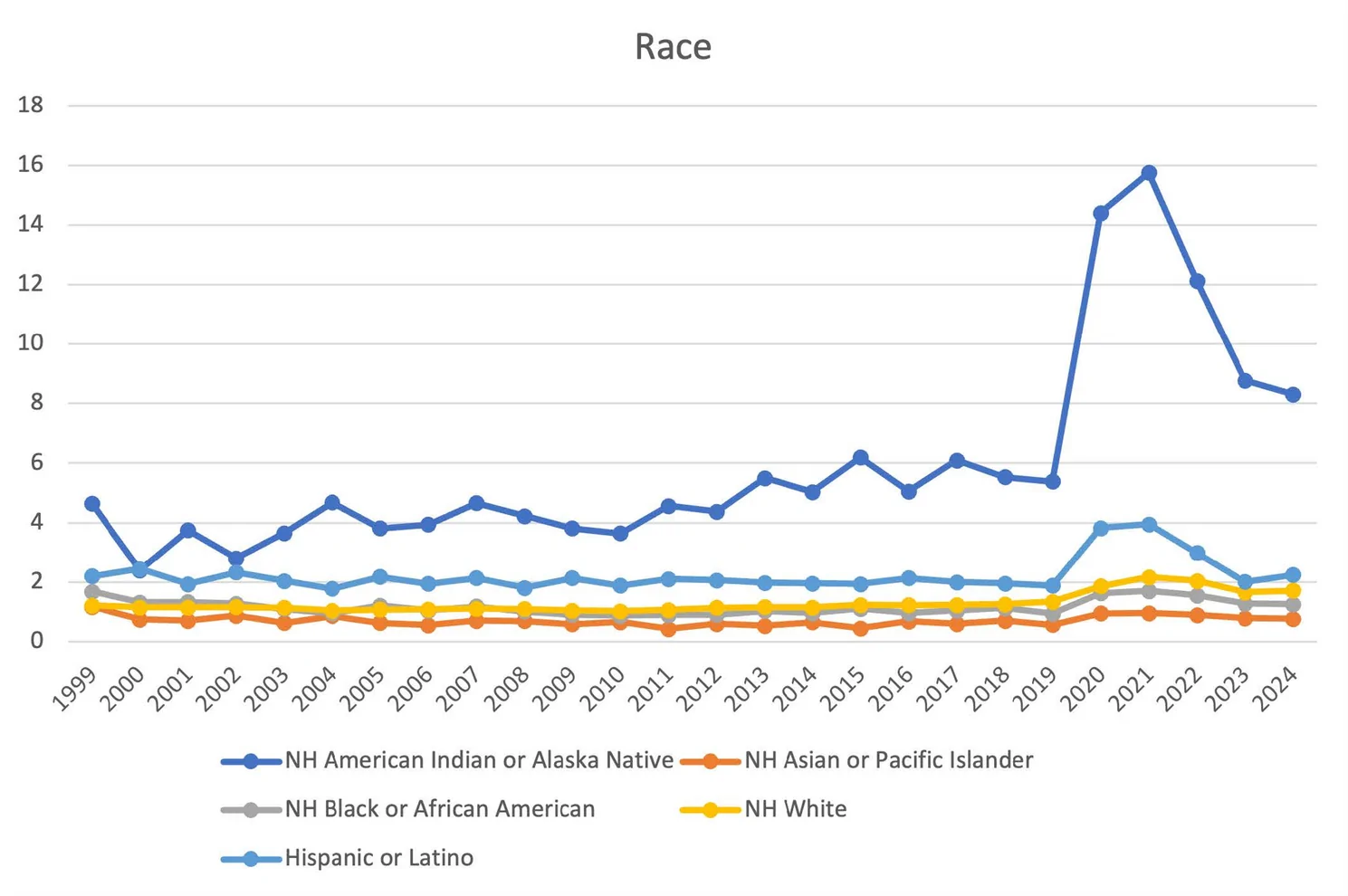
Race and ethnicity-stratified age-adjusted mortality rates (per 100,000) for deaths with co-mention of chronic liver disease and pneumonia among U.S. adults aged ≥ 25 years, 1999–2024

Non-Hispanic White persons accounted for 56,039 deaths (68.54%), Hispanic or Latino persons for 13,466 (16.47%), and non-Hispanic Black persons for 7,527 (9.21%) after reconciling the overlapping CDC releases (Table S1). AI/AN persons had the highest AAMR throughout most of the period: 4.65 in 1999, 15.75 in 2021, and 8.31 in 2024. Their 2018–2021 APC was 42.72% (95% CI, 5.88%–92.39%), followed by a 2021–2024 APC of − 23.08% (95% CI, − 32.91% to − 11.82%) (Fig. 3; Tables S3 and S5).

### Geographic patterns and urbanization

**Fig. 4:**
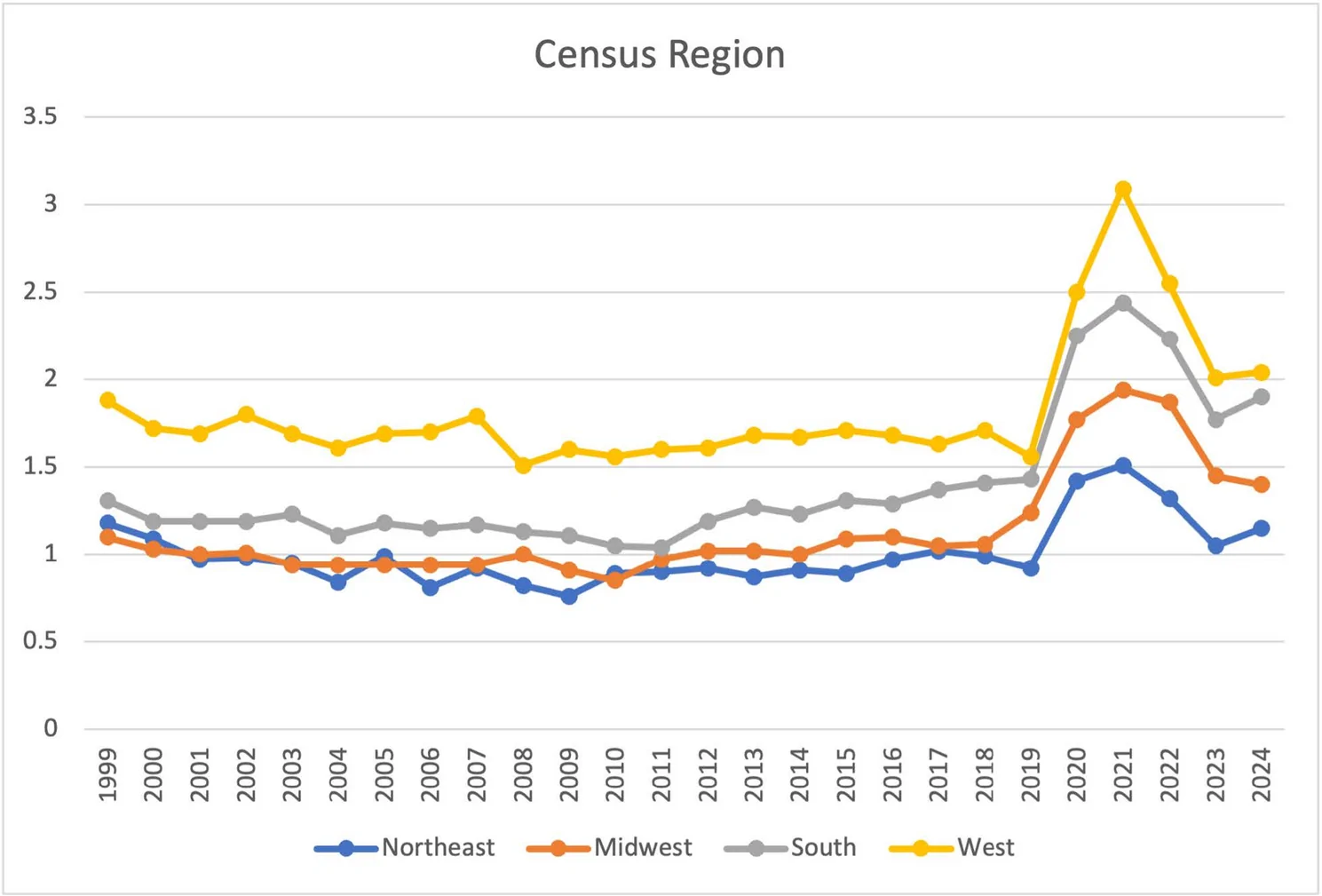
Census region-stratified age-adjusted mortality rates (per 100,000) for deaths with co-mention of chronic liver disease and pneumonia among U.S. adults aged ≥ 25 years, 1999–2024

The South contributed the largest number of deaths (31,588; 38.64%), followed by the West (24,226; 29.63%), Midwest (14,802; 18.10%), and Northeast (11,143; 13.63%). In 2024, the AAMR was highest in the West (2.04), followed by the South (1.90), Midwest (1.40), and Northeast (1.15) (Fig. 4; Tables S1, S3, and S6). New Mexico had the highest period-specific state AAMR in both 1999–2020 (2.90) and 2021–2024 (5.62) (Tables S9–S10).

**Fig. 5:**
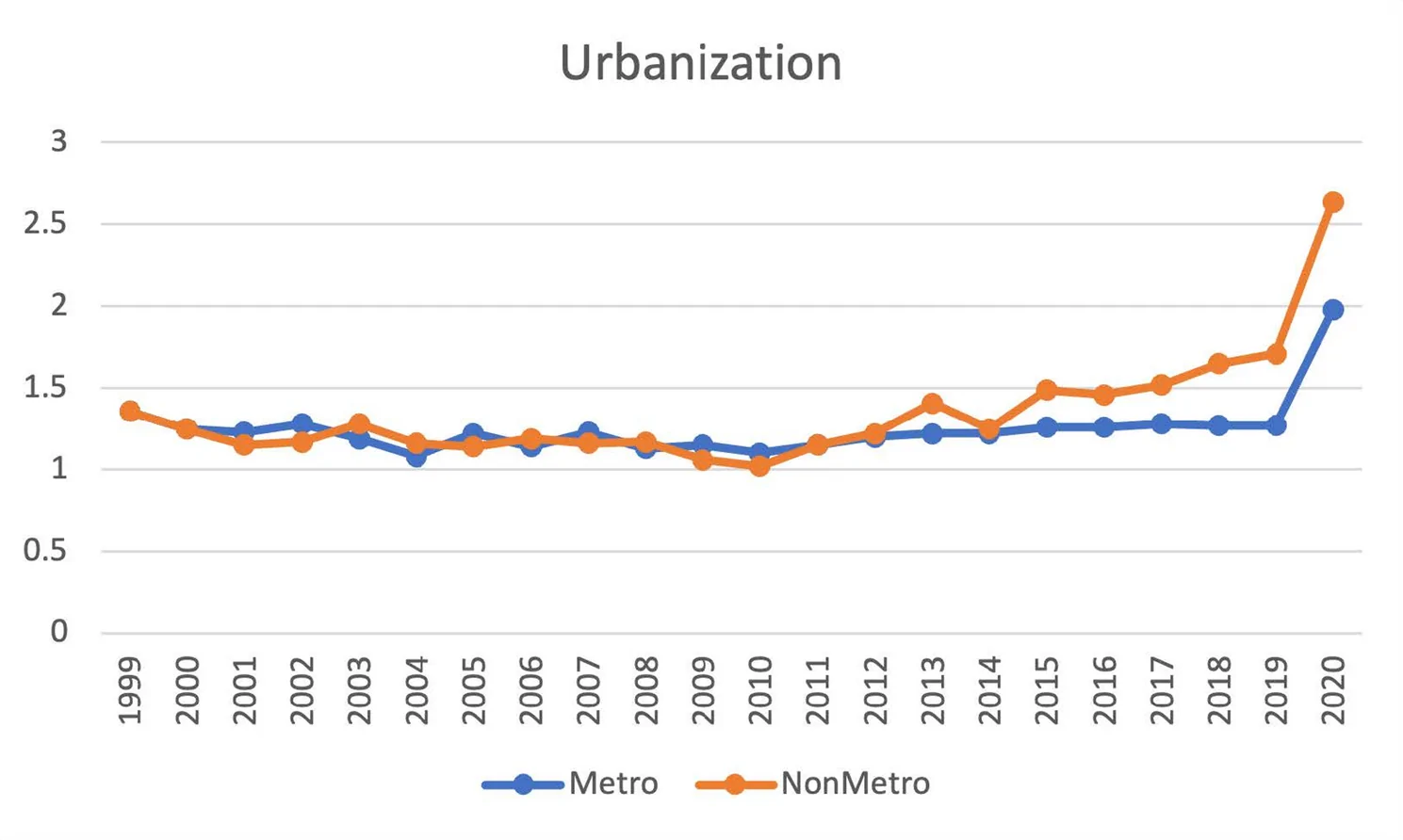
Metropolitan and non-metropolitan age-adjusted mortality rates (per 100,000) for deaths with co-mention of chronic liver disease and pneumonia among U.S. adults aged ≥ 25 years, 1999–2020

Urbanization data were available only for 1999–2020. Of 61,249 deaths in that analysis, 50,637 (82.67%) occurred among residents of metropolitan counties and 10,612 (17.33%) among residents of non-metropolitan counties. The 1999–2020 AAPC was 1.98% (95% CI, 0.81%–3.16%; *p* = 0.001) for metropolitan areas and 3.27% (95% CI, 1.79%–4.77%; *p* < 0.001) for non-metropolitan areas (Fig. 5; Tables S1, S3, and S7).

### Place of death

**Fig. 6:**
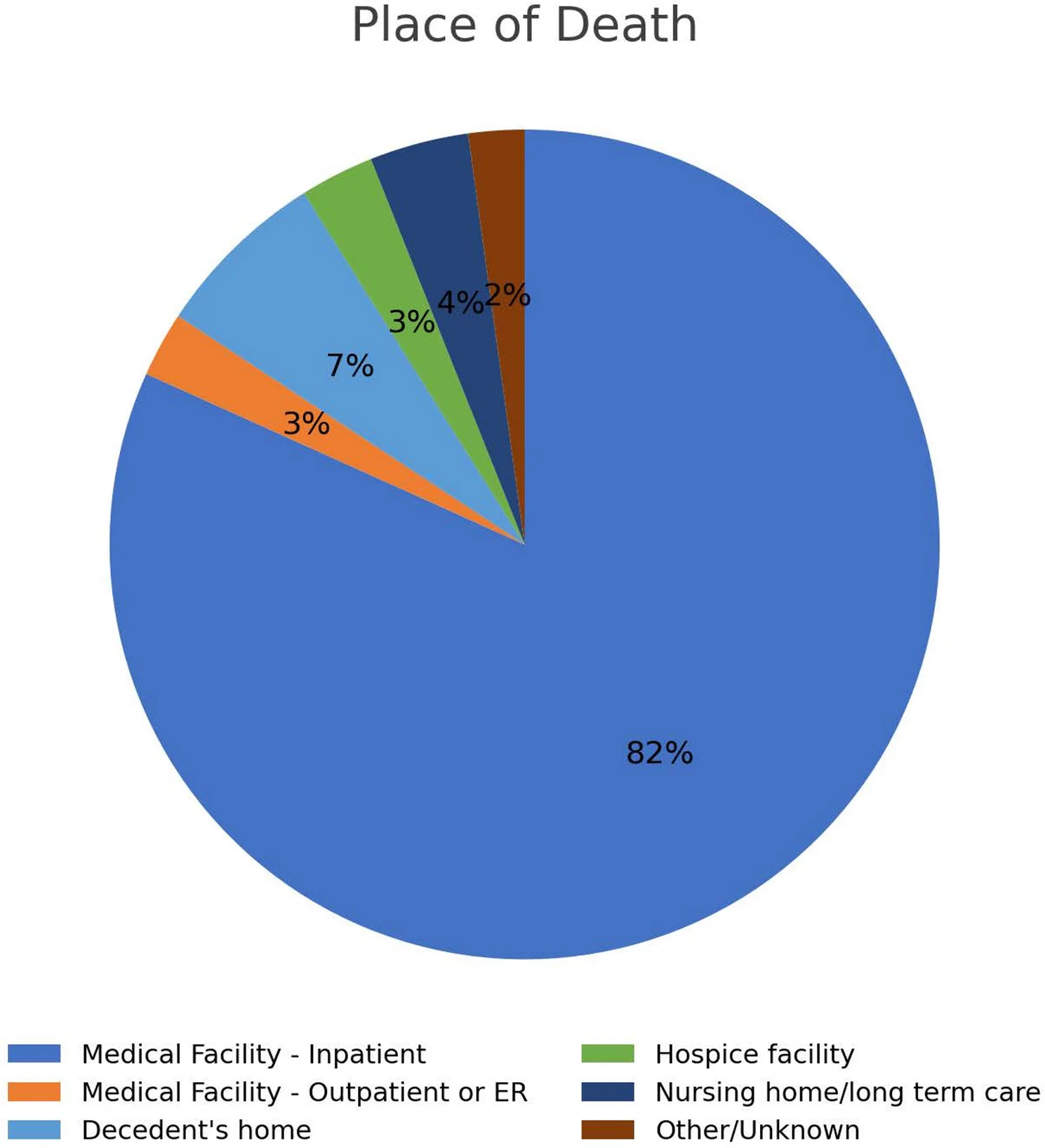
Distribution of place of death among deaths with co-mention of chronic liver disease and pneumonia, United States, 1999–2024

Most deaths occurred in an inpatient medical facility (66,833; 81.74%), followed by the decedent’s home (5,555; 6.79%), a nursing home or long-term care facility (3,157; 3.86%), hospice (2,357; 2.88%), and an outpatient medical facility or emergency department (2,075; 2.54%). Other, unknown, dead-on-arrival, suppressed, or unclassified records comprised 1,782 deaths (2.18%) (Fig. 6; Tables S1 and S8). These data describe the recorded location of death and do not identify pneumonia severity, admission indication, or hospital acquisition.

## Discussion

Co-mention of CLD and pneumonia accounted for over 81,000 deaths across our study period. Despite reported advances in both CLD and pneumonia management in recent decades [16, 17], little overall improvement in AAMR was observed, and mortality rose significantly during and after the COVID-19 pandemic, coinciding with pandemic-related disruptions to healthcare delivery. These findings suggest that existing preventive and management strategies may not have translated into substantial population-level improvements for this outcome.

CLD and pneumonia are closely associated, with advanced liver disease increasing susceptibility to respiratory infections and worsening infection-related outcomes. As described in the Background, cirrhosis-associated immune dysfunction, characterized by impaired hepatic immune surveillance, reduced synthesis of innate immune proteins, and defective phagocytic activity, plausibly underlies this relationship; bacterial infections occur in approximately 25%-50% of hospitalized patients with cirrhosis [6]. Disruption of the gut-liver-lung axis, through intestinal dysbiosis, impaired mucosal barrier integrity, and increased bacterial translocation, may further promote systemic inflammation and pulmonary infection susceptibility [18]. Alcohol consumption, a major cause of chronic liver disease and cirrhosis worldwide, may additionally contribute to this association: beyond promoting hepatic injury, alcohol impairs pulmonary host defenses and has been associated with a dose-dependent increase in community-acquired pneumonia risk, with alcohol-use disorders conferring more than an eightfold increased risk of infection [19, 20]. Malnutrition, affecting approximately 20% of patients with compensated cirrhosis and up to 50% of those with decompensated disease, is commonly accompanied by sarcopenia and frailty, and has been associated with prolonged hospitalization, increased readmission, and higher in-hospital mortality among patients hospitalized with community-acquired pneumonia [21–23].

A marked increase in mortality was observed from 2018 onward, peaking in 2021, coinciding with the COVID-19 pandemic. Several factors may plausibly have contributed to this pattern, though this study cannot establish their individual causal roles. During the pandemic, an estimated 41% of U.S. adults reported delaying or avoiding medical care, and hospital admissions and emergency department visits for non-COVID conditions declined during periods of heavy strain, patterns that may have reduced timely access to care for patients with CLD. Concurrent healthcare system strain, including staffing shortages and critical care capacity limitations, may have further disrupted care for patients with CLD and pneumonia [24]. Beyond these systemic disruptions, the pathology of COVID-19 itself may have independently amplified pneumonia-related mortality: a Canadian cohort study found non-COVID community-acquired pneumonia during the pandemic was associated with a 61% increase in hospital mortality and higher rates of ICU admission, and CLD patients infected with SARS-CoV-2 have been reported to have elevated risk of severe pneumonia, respiratory failure, and multiorgan failure, particularly with cirrhosis [25, 26]. Mortality remained elevated relative to pre-pandemic decades even after the acute phase had ended, possibly reflecting lingering effects of pandemic-related care delays; 17.6% of older adults who delayed care during the pandemic reported subsequent negative health consequences [27]. Collectively, pandemic-related healthcare disruption and the direct effects of COVID-19 infection are plausible contributors to this sustained elevation in mortality; however, this ecological, retrospective design cannot distinguish these from other possible explanations, including changes in death-certificate coding practices over time or shifts in the underlying prevalence of chronic liver disease, and cannot establish causation. This pattern persisted even after excluding deaths with a co-listed U07.1 code and when applying broader CLD and pneumonia code definitions, suggesting it was not solely attributable to COVID-19 mortality coding or the narrower primary case definition.

Sex-based differences were also observed. Although males consistently exhibited a higher mortality burden throughout our study period, females demonstrated a significant overall increase in mortality rates. The greater disease burden in males may reflect increased exposure to major risk factors for both CLD and pneumonia. Males are more likely than females to consume alcohol (68% vs. 64%), often in greater quantities, and to use tobacco products (24.5% vs. 13.9%) [28, 29], both of which are established risk factors for chronic liver disease progression and respiratory infections [30–32]. However, the increasing rates in mortality in females may be attributed to the gap in alcohol consumption rates decreasing between sexes [28], consistent with recent national data demonstrating a more rapid increase in mortality from alcohol-related liver disease among females [33], which may point to a narrowing sex disparity in alcohol-related risk factors.

Mortality burden generally increased with age through the 65–74 and 75–84-year groups; however, consistent with our findings, the oldest-old (85+) group’s rate was consistently lower than these younger older-adult groups throughout the study period, a pattern that may partly reflect survivorship, whereby individuals who reach advanced age without dying of this comorbidity are, by definition, less likely to do so. This overall pattern is consistent with known age-related biological vulnerabilities up to a point, although this study cannot establish the individual-level contribution of aging per se. Aging has been associated with a weaker immune system, with age-related immunosenescence and inflammaging impairing host immune responses and increasing susceptibility to respiratory infections and adverse outcomes [34]. Additionally, aging-related alterations in pulmonary immune function may increase susceptibility to pneumonia by facilitating bacterial invasion, impairing pathogen clearance, and prolonging recovery following infection [35]. Aging has also been associated with increased vulnerability to liver injury, enhanced fibrotic responses, and poorer outcomes across multiple chronic liver diseases, which may further contribute to the elevated mortality burden observed among older adults [36].

Our race-stratified analysis revealed that NH White and NH Black individuals accounted for the greatest number of deaths, likely relating to their greater proportion in the U.S. population [37], while AI/AN individuals exhibited the greatest mortality rates throughout our study. Several factors may plausibly contribute to this disparity, though this study cannot directly attribute it to any single cause. AI/AN populations have a higher reported smoking rate than almost every other ethnic group (34.9% as of 2020) [29], a risk factor for both CLD and pneumonia [31, 32], and CLD is already a leading cause of death among AI/AN populations [38], which may place this group at elevated risk for mortality involving co-mention of CLD and pneumonia. Barriers to healthcare access may further compound this risk: rural AI/AN populations experience substantial barriers to care, with nearly one-fifth of individuals without access to Indian Health Service care reporting delayed or forgone healthcare, and transportation difficulties, limited appointment availability, and competing time commitments identified as major contributors to delayed healthcare utilization in these communities [39].

Regional disparities were observed, with the West consistently exhibiting the highest mortality rates, followed by the South. The elevated burden in the West may partially reflect the greater representation of AI/AN populations, who had the highest mortality rates overall, as approximately 41–46% of the U.S. AI/AN population resides in the West [40]. Additionally, the elevated mortality burden observed in the West and South is consistent with prior geospatial analyses demonstrating significant clustering of liver disease-related mortality within these regions, suggesting a higher underlying burden of chronic liver disease and its associated risk factors [41].

Urbanization-related disparities were evident, with non-metropolitan areas exhibiting higher mortality rates and a greater overall increase in mortality than metropolitan regions. This disparity may partly reflect a greater burden of chronic disease risk factors in rural populations, including obesity, a known contributor to chronic liver disease progression, though these factors were not directly measured in this study [42]. Access to healthcare may also be more limited in rural communities due to physician shortages, greater travel distances to healthcare facilities, and transportation barriers; for patients with chronic liver disease, these challenges may delay diagnosis, disease monitoring, and treatment of respiratory infections, potentially increasing the risk of severe pneumonia and adverse outcomes [43].

Most deaths occurred in inpatient medical facilities. While this may in part reflect disease severity, place of death does not directly indicate pneumonia acquisition setting or severity at presentation, and the predominance of inpatient deaths may also reflect broader patterns of end-of-life care among patients with advanced chronic liver disease. This finding is consistent with the substantial inpatient burden of chronic liver disease, which is characterized by increasing hospitalization rates, longer hospital stays, and higher readmission rates than several other major chronic diseases [44]. Prolonged healthcare exposure may further increase vulnerability to hospital-acquired infections, including pneumonia, for which risk increases with duration of hospitalization and is associated with considerable mortality [45].

### Limitations

This study has several limitations. First, death certificate data are subject to misclassification and variable coding accuracy across certifiers, jurisdictions, and time. Second, our co-mention case definition cannot establish temporality, severity, or causality between CLD and pneumonia; findings reflect co-occurrence on death certificates rather than clinical incidence in a confirmed CLD cohort. Third, restricting CLD to K70, K73, and K74 excludes viral hepatitis and MASLD/NAFLD, limiting generalizability, though sensitivity analyses using broader definitions showed consistent trends. Fourth, as an ecological analysis, this study cannot adjust for individual-level factors such as disease severity, treatment, vaccination, substance use, or socioeconomic status, and subgroup differences should not be interpreted as individual-level risk. Fifth, despite a sensitivity analysis excluding deaths with a co-listed U07.1 code, residual confounding from pandemic-era coding practices and healthcare-seeking behavior cannot be fully excluded. Sixth, small case counts led to suppressed or unreliable estimates in some strata; the 25–34-year group was excluded entirely, and Native Hawaiian/Other Pacific Islander counts required proration across affected years to estimate this subgroup’s contribution to the combined NH Asian or Pacific Islander category for 2021–2024. Because this proration assumed an even distribution of suppressed deaths across years rather than reflecting the true annual counts, it could smooth over a genuine spike or decline within that period, potentially biasing the estimated magnitude, timing, or statistical significance of the joinpoint segments for this subgroup specifically; findings for the NH Asian or Pacific Islander category should therefore be interpreted with added caution relative to other race and ethnicity groups. Seventh, harmonizing CDC WONDER’s bridged-race and single-race classifications across the study period may introduce minor inconsistencies. Eighth, urbanization data were only available through 2020. Ninth, race and ethnicity are recorded by funeral directors or next of kin rather than self-reported, risking misclassification, particularly among AI/AN and multiracial populations. Finally, place of death does not indicate where pneumonia was acquired, its severity, or the clinical course leading to death.

### Clinical Implications

These findings suggest several potential public health considerations, which warrant further investigation rather than representing conclusions directly demonstrated by this study. The persistence of elevated mortality well beyond the acute phase of the COVID-19 pandemic raises the possibility that risk among patients with CLD has not returned to pre-pandemic levels, a hypothesis that could inform continued clinical attention to pneumonia in this population. The disproportionate and sustained mortality burden observed among AI/AN individuals and residents of non-metropolitan areas highlights these as priority populations for improving timely access to care, including addressing transportation and healthcare workforce barriers in underserved regions. The narrowing sex disparity, driven by a significant rise in mortality among females, suggests that sex-specific risk assumptions in CLD management may warrant reassessment, with increased attention to alcohol-related liver disease trends in women. Finally, given that deaths with co-mention of CLD and pneumonia predominantly occurred in inpatient settings, it may be worth investigating whether earlier outpatient recognition of pneumonia symptoms in patients with CLD could reduce progression to fatal illness, though this study’s death-certificate design cannot test this directly.

### Conclusion

In this national analysis of U.S. death certificates from 1999 to 2024, mortality with co-mention of chronic liver disease and pneumonia rose sharply during the COVID-19 pandemic and, despite a subsequent decline, remained elevated relative to pre-pandemic levels through 2024, a pattern that persisted across sensitivity analyses using alternative case definitions and COVID-19 exclusion criteria. Substantial disparities were observed by sex, age, race and ethnicity, region, and urbanization, with AI/AN individuals and residents of non-metropolitan areas experiencing a disproportionate and sustained mortality burden. These findings reflect patterns of co-occurrence on death certificates rather than a confirmed causal relationship between chronic liver disease and pneumonia mortality, and should be interpreted within the context of the limitations inherent to death certificate-based, ecological analysis. Continued surveillance is needed to determine whether the sustained post-pandemic elevation in mortality represents a lasting shift in disease burden, and targeted public health efforts may be warranted to address the populations identified at greatest and most persistent risk.

## Supplementary Information

Below is the link to the electronic supplementary material.

Supplementary Material 1

## Data Availability

The datasets analyzed during the current study are publicly available from the CDC WONDER Multiple Cause of Death databases (https://wonder.cdc.gov/mcd.html and https://wonder.cdc.gov/mcd-icd10-expanded.html).

## Abbreviations

AAMR: Age–Adjusted Mortality Rate
AAPC: Average Annual Percent Change
AI/AN: American Indian or Alaska Native
APC: Annual Percent Change
CDC: Centers for Disease Control and Prevention
CI: Confidence Interval
CLD: Chronic Liver Disease
COVID: 19–Coronavirus Disease 2019
ICD: 10–International Classification of Diseases, Tenth Revision
MASLD/NAFLD: Metabolic Dysfunction–Associated Steatotic Liver Disease/Non–Alcoholic Fatty Liver Disease
NCHS: National Center for Health Statistics
NH: Non–Hispanic
SARS: CoV–2–Severe Acute Respiratory Syndrome Coronavirus 2
STROBE: Strengthening the Reporting of Observational Studies in Epidemiology
U.S.: United States
WONDER: Wide–Ranging Online Data for Epidemiologic Research
