# Supplementary material for: Temporal trends and demographic disparities in mortality with co-mention of chronic liver disease and pneumonia in the United States, 1999–2024
Source: BMC Gastroenterol. 2026 Oct 1;26:623. doi: 10.1186/s12876-026-05402-3 (PMC13647610; doi:10.1186/s12876-026-05402-3)
Supplement: Supplementary file 1 [file 12876_2026_5402_MOESM1_ESM.docx]

# **Supplementary Tables**

**Temporal Trends and Demographic Disparities in Mortality with Co-Mention of Chronic Liver Disease and Pneumonia in the United States, 1999–2024**

*Tables S1–S11. All figures derived from CDC WONDER Multiple Cause of Death files (1999–2020 bridged-race; 2018–2024 single-race), reconciled as described in the Methods. Rates are per 100,000 population. AAMR = age-adjusted mortality rate; CI = 95% confidence interval.*

**Table S1. Absolute number of deaths and percent of total, by subgroup, 1999–2024.**

*Supplementary reproduction of the main-text Table 1 summary, provided for completeness alongside the detailed year-by-year and trend tables below (Tables S2–S8).*

| **Variable** | **Deaths** | **% of Total** |
| --- | --- | --- |
| Overall | 81759 | 100% |
| **Sex** | | |
| Male | 50682 | 61.98% |
| Female | 31077 | 38.01% |
| **Race/Ethnicity** | | |
| NH White | 56039 | 68.54% |
| NH Black or African American | 7527 | 9.20% |
| NH American Indian or Alaska Native | 2403 | 2.93% |
| NH Asian or Pacific Islander | 1825 | 2.23% |
| NH More than one race | 218 | 0.26% |
| Hispanic or Latino | 13466 | 16.47% |
| **Age Groups** | | |
| Young adults (25-34 years) | 1434 | 1.75% |
| Adults (35-44 years) | 6091 | 7.44% |
| Middle-aged adults (45-54 years) | 16899 | 20.66% |
| Older adults (55-64 years) | 24555 | 30.03% |
| Young-old adults (65-74 years) | 18810 | 23.00% |
| Middle-old adults (75-84 years) | 10543 | 12.89% |
| Oldest-old adults (85+ years) | 3427 | 4.19% |
| **Census Region** | | |
| Northeast | 11143 | 13.62% |
| Midwest | 14802 | 18.10% |
| South | 31588 | 38.63% |
| West | 24226 | 29.63% |
| **Urbanization** | | |
| Metropolitan | 50637 | 82.68% |
| Non-metropolitan | 10612 | 17.32% |
| **Place of Death** | | |
| Medical Facility - Inpatient | 66833 | 81.74% |
| Medical Facility - Outpatient or ER | 2075 | 2.53% |
| Decedent's Home | 5555 | 6.79% |
| Hospice Facility | 2357 | 2.88% |
| Nursing Home / Long-term Care | 3157 | 3.86% |
| Other / Unknown | 1782 | 2.18% |

*NH = Non-Hispanic. Race/ethnicity categories may not sum to the overall total due to a small number of deaths with race not stated; the NH Asian or Pacific Islander total for 2021–2024 includes a prorated estimate of suppressed Native Hawaiian/Other Pacific Islander deaths (see Table 1 footnote). Urbanization percentages use the 1999–2020 total (N = 61,249) as the denominator. Place of death “Other/Unknown” combines Dead on Arrival, Status Unknown, Place of Death Unknown, Other, and suppressed/unclassifiable records (see Table S8 for the detailed breakdown).*

**Table S2. Overall and sex-stratified age-adjusted mortality rates, 1999–2024.**

*AAMR (95% CI) per 100,000 population, by year and sex.*

| **Year** | **Overall AAMR (95% CI)** | **Male AAMR (95% CI)** | **Female AAMR (95% CI)** |
| --- | --- | --- | --- |
| 1999 | 1.36 (1.31–1.42) | 1.99 (1.89–2.08) | 0.82 (0.76–0.88) |
| 2000 | 1.24 (1.19–1.29) | 1.81 (1.72–1.91) | 0.73 (0.68–0.78) |
| 2001 | 1.22 (1.17–1.27) | 1.74 (1.65–1.83) | 0.73 (0.68–0.78) |
| 2002 | 1.24 (1.19–1.29) | 1.75 (1.66–1.84) | 0.82 (0.77–0.88) |
| 2003 | 1.23 (1.18–1.28) | 1.74 (1.65–1.82) | 0.78 (0.72–0.83) |
| 2004 | 1.10 (1.06–1.15) | 1.55 (1.47–1.63) | 0.77 (0.71–0.82) |
| 2005 | 1.20 (1.16–1.25) | 1.69 (1.61–1.78) | 0.76 (0.71–0.81) |
| 2006 | 1.13 (1.08–1.17) | 1.62 (1.54–1.70) | 0.75 (0.70–0.80) |
| 2007 | 1.21 (1.16–1.26) | 1.71 (1.63–1.79) | 0.77 (0.72–0.82) |
| 2008 | 1.14 (1.10–1.19) | 1.58 (1.50–1.65) | 0.74 (0.69–0.79) |
| 2009 | 1.11 (1.07–1.16) | 1.52 (1.44–1.60) | 0.73 (0.68–0.78) |
| 2010 | 1.10 (1.06–1.15) | 1.48 (1.41–1.56) | 0.72 (0.67–0.77) |
| 2011 | 1.16 (1.11–1.20) | 1.55 (1.48–1.63) | 0.79 (0.74–0.84) |
| 2012 | 1.19 (1.15–1.24) | 1.61 (1.53–1.69) | 0.83 (0.77–0.88) |
| 2013 | 1.24 (1.19–1.28) | 1.62 (1.54–1.70) | 0.92 (0.86–0.97) |
| 2014 | 1.22 (1.17–1.26) | 1.58 (1.50–1.65) | 0.91 (0.85–0.96) |
| 2015 | 1.29 (1.24–1.33) | 1.67 (1.59–1.74) | 0.95 (0.89–1.01) |
| 2016 | 1.29 (1.25–1.34) | 1.67 (1.59–1.74) | 0.96 (0.91–1.02) |
| 2017 | 1.31 (1.26–1.35) | 1.66 (1.58–1.73) | 1.00 (0.95–1.06) |
| 2018 | 1.32 (1.28–1.37) | 1.65 (1.58–1.73) | 1.05 (0.99–1.11) |
| 2019 | 1.34 (1.29–1.38) | 1.67 (1.60–1.75) | 1.03 (0.97–1.08) |
| 2020 | 2.07 (2.01–2.13) | 2.64 (2.55–2.73) | 1.55 (1.48–1.62) |
| 2021 | 2.32 (2.26–2.38) | 2.85 (2.76–2.95) | 1.85 (1.78–1.93) |
| 2022 | 2.06 (2.00–2.12) | 2.59 (2.50–2.68) | 1.60 (1.54–1.68) |
| 2023 | 1.65 (1.60–1.70) | 1.99 (1.91–2.07) | 1.32 (1.26–1.39) |
| 2024 | 1.69 (1.64–1.74) | 2.10 (2.02–2.18) | 1.35 (1.29–1.41) |

**Table S3. Annual percent change (APC) in age-adjusted mortality rates, by subgroup, 1999–2024.**

*Full Joinpoint regression results (APC, 95% CI, and p-value) for all subgroups analyzed. These results are also presented in Table 2 of the main text and are reproduced here for completeness alongside the other supplementary tables.*

| **Subgroup** | **Year Interval** | **APC (95% CI)** | **p-Value** |
| --- | --- | --- | --- |
| **Overall** |  |  |  |
|  | 1999–2018 | 0.33 (-0.26 to 0.91) | 0.258 |
|  | 2018–2021 | 23.25* (5.70 to 43.71) | 0.010 |
|  | 2021–2024 | -11.78* (-18.91 to -4.02) | 0.006 |
| **Female** |  |  |  |
|  | 1999–2009 | -0.49 (-1.79 to 0.83) | 0.439 |
|  | 2009–2018 | 3.52* (1.83 to 5.24) | <0.001 |
|  | 2018–2021 | 20.79* (8.54 to 34.41) | 0.002 |
|  | 2021–2024 | -10.76* (-15.19 to -6.09) | <0.001 |
| **Male** |  |  |  |
|  | 1999–2018 | -0.61 (-1.22 to 0.00) | 0.051 |
|  | 2018–2021 | 22.91* (3.22 to 46.36) | 0.023 |
|  | 2021–2024 | -12.15* (-18.71 to -5.07) | 0.003 |
| **Northeast** |  |  |  |
|  | 1999–2008 | -3.31* (-5.23 to -1.35) | 0.003 |
|  | 2008–2018 | 1.81 (-0.21 to 3.86) | 0.075 |
|  | 2018–2021 | 14.92 (-6.24 to 40.85) | 0.166 |
|  | 2021–2024 | -10.43* (-17.73 to -2.48) | 0.015 |
| **Midwest** |  |  |  |
|  | 1999–2009 | -1.32* (-2.61 to -0.02) | 0.046 |
|  | 2009–2018 | 2.06* (0.33 to 3.82) | 0.023 |
|  | 2018–2021 | 22.44* (7.86 to 39.00) | 0.004 |
|  | 2021–2024 | -12.25* (-17.17 to -7.04) | <0.001 |
| **South** |  |  |  |
|  | 1999–2010 | -1.14 (-2.39 to 0.13) | 0.076 |
|  | 2010–2018 | 2.98* (0.59 to 5.44) | 0.018 |
|  | 2018–2021 | 20.91* (5.61 to 38.43) | 0.009 |
|  | 2021–2024 | -10.06* (-15.14 to -4.67) | 0.002 |
| **West** |  |  |  |
|  | 1999–2018 | -0.53* (-1.01 to -0.06) | 0.030 |
|  | 2018–2021 | 23.27* (7.86 to 40.88) | 0.004 |
|  | 2021–2024 | -13.60* (-18.88 to -7.97) | <0.001 |
| **Metro** |  |  |  |
|  | 1999–2018 | -0.16 (-0.69 to 0.36) | 0.522 |
|  | 2018–2020 | 24.74* (10.65 to 40.62) | 0.001 |
| **Non-Metro** |  |  |  |
|  | 1999–2010 | -1.29* (-2.45 to -0.12) | 0.033 |
|  | 2010–2018 | 4.80* (2.60 to 7.05) | <0.001 |
|  | 2018–2020 | 24.82* (9.96 to 41.68) | 0.002 |
| **NH American Indian or Alaska Native** |  |  |  |
|  | 1999–2018 | 2.78* (1.41 to 4.17) | <0.001 |
|  | 2018–2021 | 42.72* (5.88 to 92.39) | 0.022 |
|  | 2021–2024 | -23.08* (-32.91 to -11.82) | <0.001 |
| **NH Asian or Pacific Islander** |  |  |  |
|  | 1999–2011 | -4.68* (-8.06 to -1.18) | 0.012 |
|  | 2011–2024 | 4.19* (1.76 to 6.69) | 0.002 |
| **NH Black or African American** |  |  |  |
|  | 1999–2010 | -4.35* (-6.15 to -2.51) | <0.001 |
|  | 2010–2018 | 2.00 (-1.65 to 5.78) | 0.264 |
|  | 2018–2021 | 17.38 (-8.46 to 50.52) | 0.190 |
|  | 2021–2024 | -10.64* (-19.25 to -1.12) | 0.032 |
| **NH White** |  |  |  |
|  | 1999–2009 | -1.15* (-2.20 to -0.09) | 0.036 |
|  | 2009–2018 | 1.93* (0.41 to 3.47) | 0.016 |
|  | 2018–2021 | 19.93* (8.31 to 32.80) | 0.002 |
|  | 2021–2024 | -8.74* (-13.06 to -4.21) | 0.001 |
| **Hispanic or Latino** |  |  |  |
|  | 1999–2018 | -0.54 (-1.40 to 0.33) | 0.207 |
|  | 2018–2021 | 27.08* (3.54 to 55.96) | 0.024 |
|  | 2021–2024 | -20.99* (-28.12 to -13.15) | <0.001 |
| **35–44 years** |  |  |  |
|  | 1999–2009 | -4.30* (-5.93 to -2.64) | <0.001 |
|  | 2009–2018 | 2.73* (0.17 to 5.34) | 0.038 |
|  | 2018–2021 | 27.00* (2.21 to 57.80) | 0.033 |
|  | 2021–2024 | -10.33* (-18.12 to -1.80) | 0.022 |
| **45–54 years** |  |  |  |
|  | 1999–2018 | -0.43 (-0.89 to 0.03) | 0.065 |
|  | 2018–2021 | 19.00* (3.06 to 37.39) | 0.020 |
|  | 2021–2024 | -14.21* (-19.81 to -8.22) | <0.001 |
| **55–64 years** |  |  |  |
|  | 1999–2018 | 2.24* (1.58 to 2.91) | <0.001 |
|  | 2018–2021 | 16.80 (-0.12 to 36.58) | 0.052 |
|  | 2021–2024 | -14.53* (-20.69 to -7.89) | <0.001 |
| **65–74 years** |  |  |  |
|  | 1999–2009 | -2.88* (-4.66 to -1.06) | 0.004 |
|  | 2009–2018 | 3.09* (0.76 to 5.48) | 0.013 |
|  | 2018–2021 | 23.43* (6.07 to 43.64) | 0.010 |
|  | 2021–2024 | -11.63* (-17.38 to -5.47) | 0.001 |
| **75–84 years** |  |  |  |
|  | 1999–2010 | -4.00* (-5.48 to -2.50) | <0.001 |
|  | 2010–2018 | 3.11 (-0.11 to 6.43) | 0.057 |
|  | 2018–2021 | 19.13 (-1.49 to 44.06) | 0.068 |
|  | 2021–2024 | -6.45 (-13.35 to 0.99) | 0.083 |
| **85+ years** |  |  |  |
|  | 1999–2018 | -2.05* (-3.07 to -1.01) | 0.001 |
|  | 2018–2021 | 28.08 (-4.38 to 71.58) | 0.092 |
|  | 2021–2024 | -1.84 (-13.09 to 10.86) | 0.752 |

** Indicates APC significantly different from zero at α = 0.05.*

**Table S4. Age-specific crude mortality rates, 1999–2024.**

*Crude mortality rate (95% CI) per 100,000 population, by year and age group. Age-adjustment is not applicable within a single age stratum.*

| **Year** | **25-34** | **35-44** | **45-54** | **55-64** | **65-74** | **75-84** | **85+** |
| --- | --- | --- | --- | --- | --- | --- | --- |
| 1999 | 0.09 (0.06–0.12) | 0.67 (0.59–0.74) | 1.58 (1.45–1.71) | 2.14 (1.95–2.33) | 2.76 (2.52–3.00) | 3.04 (2.73–3.35) | 2.26 (1.83–2.77) |
| 2000 | 0.06 (0.04–0.09) | 0.54 (0.47–0.61) | 1.41 (1.29–1.53) | 1.84 (1.67–2.01) | 2.56 (2.32–2.79) | 3.37 (3.05–3.70) | 2.45 (1.98–2.92) |
| 2001 | 0.06 (0.04–0.09) | 0.57 (0.50–0.64) | 1.41 (1.29–1.53) | 1.83 (1.66–2.00) | 2.55 (2.32–2.78) | 2.78 (2.49–3.07) | 2.37 (1.91–2.82) |
| 2002 | 0.07 (0.05–0.10) | 0.51 (0.44–0.57) | 1.44 (1.32–1.55) | 2.00 (1.83–2.17) | 2.50 (2.27–2.73) | 2.89 (2.60–3.19) | 3.07 (2.55–3.59) |
| 2003 | 0.06 (0.04–0.09) | 0.48 (0.42–0.55) | 1.46 (1.34–1.57) | 1.86 (1.70–2.02) | 2.54 (2.31–2.77) | 2.81 (2.52–3.10) | 2.60 (2.12–3.07) |
| 2004 | Unreliable | 0.44 (0.38–0.51) | 1.48 (1.36–1.60) | 1.76 (1.61–1.91) | 2.22 (2.00–2.43) | 2.51 (2.24–2.78) | 2.07 (1.67–2.53) |
| 2005 | Unreliable | 0.46 (0.40–0.53) | 1.56 (1.45–1.68) | 2.03 (1.87–2.19) | 2.49 (2.26–2.71) | 2.33 (2.07–2.59) | 2.51 (2.06–2.97) |
| 2006 | Unreliable | 0.44 (0.37–0.50) | 1.55 (1.43–1.67) | 1.93 (1.78–2.08) | 2.18 (1.97–2.39) | 2.58 (2.31–2.86) | 2.20 (1.78–2.62) |
| 2007 | 0.07 (0.04–0.10) | 0.48 (0.42–0.55) | 1.55 (1.43–1.66) | 2.21 (2.05–2.37) | 2.28 (2.07–2.49) | 2.38 (2.12–2.65) | 2.24 (1.83–2.66) |
| 2008 | 0.06 (0.04–0.09) | 0.39 (0.33–0.45) | 1.47 (1.36–1.58) | 2.31 (2.15–2.47) | 1.98 (1.78–2.17) | 2.13 (1.88–2.38) | 2.10 (1.70–2.49) |
| 2009 | 0.09 (0.06–0.12) | 0.40 (0.34–0.47) | 1.54 (1.43–1.66) | 2.17 (2.01–2.32) | 1.91 (1.73–2.10) | 2.03 (1.78–2.27) | 1.96 (1.58–2.33) |
| 2010 | 0.06 (0.04–0.09) | 0.41 (0.35–0.47) | 1.35 (1.24–1.46) | 2.15 (2.00–2.30) | 2.10 (1.91–2.29) | 2.06 (1.81–2.31) | 1.97 (1.60–2.34) |
| 2011 | 0.08 (0.06–0.11) | 0.38 (0.32–0.44) | 1.49 (1.38–1.60) | 2.36 (2.20–2.51) | 2.06 (1.87–2.24) | 2.07 (1.83–2.32) | 1.67 (1.36–2.04) |
| 2012 | 0.09 (0.06–0.12) | 0.44 (0.37–0.50) | 1.46 (1.34–1.57) | 2.44 (2.28–2.59) | 2.37 (2.18–2.57) | 2.19 (1.94–2.44) | 1.56 (1.26–1.92) |
| 2013 | 0.13 (0.10–0.17) | 0.40 (0.33–0.46) | 1.55 (1.43–1.67) | 2.52 (2.37–2.68) | 2.43 (2.24–2.63) | 2.19 (1.94–2.44) | 2.00 (1.65–2.36) |
| 2014 | 0.12 (0.09–0.15) | 0.41 (0.35–0.47) | 1.47 (1.35–1.58) | 2.64 (2.48–2.80) | 2.23 (2.05–2.41) | 2.40 (2.14–2.66) | 1.70 (1.38–2.03) |
| 2015 | 0.08 (0.06–0.11) | 0.45 (0.39–0.52) | 1.44 (1.33–1.55) | 2.71 (2.55–2.87) | 2.53 (2.34–2.72) | 2.56 (2.30–2.83) | 1.99 (1.64–2.34) |
| 2016 | 0.16 (0.13–0.20) | 0.49 (0.42–0.56) | 1.33 (1.22–1.43) | 2.77 (2.61–2.93) | 2.61 (2.43–2.80) | 2.52 (2.26–2.78) | 1.61 (1.30–1.93) |
| 2017 | 0.15 (0.11–0.19) | 0.50 (0.43–0.57) | 1.36 (1.25–1.47) | 2.70 (2.54–2.85) | 2.84 (2.65–3.03) | 2.45 (2.20–2.70) | 2.06 (1.71–2.41) |
| 2018 | 0.16 (0.12–0.20) | 0.52 (0.45–0.59) | 1.41 (1.30–1.53) | 2.72 (2.56–2.88) | 2.64 (2.45–2.82) | 2.73 (2.47–3.00) | 1.94 (1.60–2.28) |
| 2019 | 0.15 (0.12–0.19) | 0.53 (0.46–0.60) | 1.37 (1.25–1.48) | 2.83 (2.67–2.99) | 2.72 (2.54–2.90) | 2.58 (2.33–2.83) | 1.94 (1.60–2.27) |
| 2020 | 0.30 (0.25–0.35) | 0.82 (0.73–0.91) | 2.09 (1.95–2.23) | 4.05 (3.86–4.24) | 4.50 (4.27–4.73) | 3.96 (3.66–4.27) | 3.27 (2.84–3.71) |
| 2021 | 0.29 (0.24–0.34) | 1.02 (0.93–1.12) | 2.39 (2.24–2.54) | 4.43 (4.23–4.63) | 5.09 (4.85–5.33) | 4.42 (4.10–4.75) | 3.48 (3.01–3.95) |
| 2022 | 0.32 (0.26–0.37) | 0.93 (0.84–1.02) | 2.04 (1.90–2.17) | 3.80 (3.61–3.98) | 4.50 (4.28–4.73) | 4.33 (4.02–4.63) | 3.53 (3.07–3.99) |
| 2023 | 0.22 (0.18–0.27) | 0.75 (0.67–0.84) | 1.66 (1.53–1.78) | 2.84 (2.68–3.00) | 3.47 (3.28–3.67) | 3.37 (3.10–3.64) | 3.39 (2.93–3.85) |
| 2024 | 0.26 (0.21–0.30) | 0.76 (0.68–0.84) | 1.54 (1.42–1.67) | 2.95 (2.78–3.11) | 3.74 (3.54–3.94) | 3.82 (3.55–4.10) | 3.54 (3.08–4.00) |

*Note: Rates for the 25–34-year age group in 2004–2006 were flagged as unreliable by CDC WONDER due to small case counts (see Methods); this age group was excluded from Joinpoint trend analysis in its entirety.*

**Table S5. Race and ethnicity-stratified age-adjusted mortality rates, 1999–2024.**

*AAMR (95% CI) per 100,000 population, by year and race/ethnicity. Race was classified using bridged-race categories for 1999–2020 and single-race categories for 2021–2024, harmonized as described in the Methods.*

| **Year** | **NH AIAN** | **NH Asian/PI** | **NH Black** | **NH White** | **Hispanic** |
| --- | --- | --- | --- | --- | --- |
| 1999 | 4.65 (3.42–6.18) | 1.17 (0.86–1.56) | 1.68 (1.49–1.88) | 1.22 (1.17–1.28) | 2.20 (1.92–2.48) |
| 2000 | 2.41 (1.54–3.58) | 0.75 (0.52–1.04) | 1.32 (1.15–1.49) | 1.15 (1.09–1.20) | 2.45 (2.15–2.75) |
| 2001 | 3.75 (2.63–5.20) | 0.71 (0.50–0.99) | 1.33 (1.16–1.50) | 1.15 (1.10–1.21) | 1.94 (1.68–2.19) |
| 2002 | 2.81 (1.96–3.91) | 0.88 (0.64–1.18) | 1.28 (1.12–1.45) | 1.16 (1.11–1.22) | 2.33 (2.06–2.60) |
| 2003 | 3.66 (2.66–4.92) | 0.63 (0.44–0.88) | 1.10 (0.96–1.25) | 1.14 (1.08–1.19) | 2.04 (1.79–2.29) |
| 2004 | 4.69 (3.49–6.16) | 0.86 (0.63–1.13) | 0.95 (0.81–1.08) | 1.04 (0.98–1.09) | 1.78 (1.55–2.00) |
| 2005 | 3.82 (2.78–5.13) | 0.63 (0.45–0.86) | 1.22 (1.07–1.38) | 1.08 (1.03–1.14) | 2.18 (1.94–2.42) |
| 2006 | 3.94 (2.88–5.25) | 0.56 (0.40–0.78) | 1.05 (0.91–1.19) | 1.09 (1.04–1.15) | 1.95 (1.73–2.17) |
| 2007 | 4.67 (3.49–6.13) | 0.70 (0.51–0.93) | 1.18 (1.04–1.33) | 1.11 (1.05–1.16) | 2.14 (1.91–2.37) |
| 2008 | 4.23 (3.23–5.43) | 0.69 (0.51–0.91) | 1.01 (0.87–1.14) | 1.10 (1.04–1.15) | 1.81 (1.60–2.02) |
| 2009 | 3.82 (2.83–5.05) | 0.59 (0.43–0.78) | 0.91 (0.78–1.03) | 1.05 (1.00–1.10) | 2.14 (1.92–2.35) |
| 2010 | 3.65 (2.69–4.84) | 0.66 (0.50–0.87) | 0.88 (0.76–1.00) | 1.02 (0.97–1.07) | 1.88 (1.68–2.08) |
| 2011 | 4.57 (3.50–5.87) | 0.43 (0.30–0.60) | 0.90 (0.78–1.02) | 1.07 (1.02–1.12) | 2.11 (1.90–2.31) |
| 2012 | 4.38 (3.38–5.59) | 0.60 (0.45–0.78) | 0.91 (0.78–1.03) | 1.14 (1.08–1.19) | 2.06 (1.86–2.26) |
| 2013 | 5.50 (4.36–6.85) | 0.53 (0.40–0.71) | 1.03 (0.91–1.16) | 1.16 (1.11–1.21) | 1.98 (1.79–2.16) |
| 2014 | 5.04 (3.98–6.30) | 0.65 (0.50–0.83) | 0.96 (0.84–1.08) | 1.15 (1.10–1.21) | 1.96 (1.78–2.14) |
| 2015 | 6.20 (4.98–7.42) | 0.45 (0.34–0.60) | 1.11 (0.98–1.24) | 1.24 (1.18–1.29) | 1.94 (1.76–2.11) |
| 2016 | 5.06 (4.04–6.27) | 0.68 (0.54–0.85) | 0.96 (0.84–1.07) | 1.23 (1.17–1.28) | 2.14 (1.96–2.33) |
| 2017 | 6.10 (4.91–7.29) | 0.60 (0.46–0.76) | 1.04 (0.92–1.16) | 1.24 (1.18–1.29) | 2.00 (1.83–2.17) |
| 2018 | 5.54 (4.42–6.65) | 0.70 (0.57–0.87) | 1.13 (1.00–1.26) | 1.26 (1.21–1.31) | 1.96 (1.79–2.13) |
| 2019 | 5.39 (4.33–6.62) | 0.57 (0.45–0.72) | 0.94 (0.83–1.06) | 1.34 (1.28–1.39) | 1.88 (1.72–2.04) |
| 2020 | 14.40 (12.59–16.21) | 0.95 (0.79–1.11) | 1.63 (1.49–1.78) | 1.86 (1.79–1.93) | 3.83 (3.60–4.06) |
| 2021 | 15.75 (13.85–17.87) | 0.96 (0.80–1.14)* | 1.70 (1.56–1.86) | 2.17 (2.10–2.25) | 3.96 (3.73–4.19) |
| 2022 | 12.12 (10.43–14.01) | 0.90 (0.75–1.08)* | 1.55 (1.40–1.70) | 2.04 (1.97–2.11) | 2.99 (2.80–3.19) |
| 2023 | 8.78 (7.39–10.37) | 0.79 (0.65–0.94)* | 1.28 (1.15–1.42) | 1.66 (1.60–1.73) | 2.01 (1.85–2.17) |
| 2024 | 8.31 (6.96–9.85) | 0.77 (0.64–0.92)* | 1.26 (1.13–1.39) | 1.71 (1.65–1.78) | 2.25 (2.10–2.42) |

** For 2021–2024, the NH Asian or Pacific Islander rate reflects the single-race “Asian” category only; Native Hawaiian/Other Pacific Islander rates were suppressed by CDC WONDER in most years due to small cell sizes and are not separately shown by year (see Table 1 footnote for the prorated death-count estimate used in aggregate totals). AIAN = American Indian or Alaska Native; PI = Pacific Islander.*

**Table S6. Census region-stratified age-adjusted mortality rates, 1999–2024.**

*AAMR (95% CI) per 100,000 population, by year and U.S. census region.*

| **Year** | **Northeast** | **Midwest** | **South** | **West** |
| --- | --- | --- | --- | --- |
| 1999 | 1.18 (1.06–1.29) | 1.10 (1.00–1.21) | 1.31 (1.22–1.40) | 1.88 (1.74–2.02) |
| 2000 | 1.09 (0.98–1.20) | 1.03 (0.93–1.13) | 1.19 (1.11–1.28) | 1.72 (1.58–1.85) |
| 2001 | 0.97 (0.87–1.07) | 1.00 (0.91–1.10) | 1.19 (1.11–1.28) | 1.69 (1.56–1.82) |
| 2002 | 0.98 (0.88–1.08) | 1.01 (0.92–1.11) | 1.19 (1.11–1.28) | 1.80 (1.67–1.93) |
| 2003 | 0.95 (0.85–1.04) | 0.94 (0.85–1.03) | 1.23 (1.15–1.32) | 1.69 (1.57–1.82) |
| 2004 | 0.84 (0.74–0.93) | 0.94 (0.84–1.03) | 1.11 (1.03–1.19) | 1.61 (1.49–1.74) |
| 2005 | 0.99 (0.89–1.09) | 0.94 (0.85–1.04) | 1.18 (1.10–1.26) | 1.69 (1.57–1.82) |
| 2006 | 0.81 (0.72–0.90) | 0.94 (0.85–1.04) | 1.15 (1.07–1.22) | 1.70 (1.58–1.82) |
| 2007 | 0.92 (0.82–1.02) | 0.94 (0.85–1.03) | 1.17 (1.09–1.24) | 1.79 (1.67–1.92) |
| 2008 | 0.82 (0.73–0.91) | 1.00 (0.91–1.09) | 1.13 (1.06–1.21) | 1.51 (1.40–1.62) |
| 2009 | 0.76 (0.67–0.84) | 0.91 (0.82–0.99) | 1.11 (1.04–1.18) | 1.60 (1.49–1.71) |
| 2010 | 0.89 (0.79–0.98) | 0.85 (0.77–0.94) | 1.05 (0.98–1.12) | 1.56 (1.45–1.68) |
| 2011 | 0.90 (0.81–0.99) | 0.97 (0.88–1.06) | 1.04 (0.97–1.12) | 1.60 (1.49–1.71) |
| 2012 | 0.92 (0.83–1.01) | 1.02 (0.92–1.11) | 1.19 (1.12–1.27) | 1.61 (1.50–1.72) |
| 2013 | 0.87 (0.78–0.95) | 1.02 (0.93–1.11) | 1.27 (1.20–1.35) | 1.68 (1.57–1.80) |
| 2014 | 0.91 (0.82–1.00) | 1.00 (0.92–1.09) | 1.23 (1.16–1.30) | 1.67 (1.56–1.78) |
| 2015 | 0.89 (0.80–0.98) | 1.09 (1.00–1.18) | 1.31 (1.24–1.39) | 1.71 (1.60–1.82) |
| 2016 | 0.97 (0.87–1.06) | 1.10 (1.01–1.19) | 1.29 (1.22–1.36) | 1.68 (1.57–1.79) |
| 2017 | 1.02 (0.92–1.11) | 1.05 (0.96–1.14) | 1.37 (1.30–1.45) | 1.63 (1.53–1.74) |
| 2018 | 0.99 (0.89–1.08) | 1.06 (0.98–1.15) | 1.41 (1.33–1.49) | 1.71 (1.60–1.82) |
| 2019 | 0.92 (0.83–1.00) | 1.24 (1.15–1.34) | 1.43 (1.36–1.51) | 1.56 (1.46–1.67) |
| 2020 | 1.42 (1.31–1.53) | 1.77 (1.65–1.88) | 2.25 (2.15–2.34) | 2.50 (2.37–2.63) |
| 2021 | 1.51 (1.40–1.63) | 1.94 (1.82–2.07) | 2.44 (2.34–2.54) | 3.09 (2.95–3.24) |
| 2022 | 1.32 (1.22–1.43) | 1.87 (1.75–1.99) | 2.23 (2.14–2.33) | 2.55 (2.42–2.68) |
| 2023 | 1.05 (0.96–1.15) | 1.45 (1.35–1.56) | 1.77 (1.69–1.85) | 2.01 (1.90–2.14) |
| 2024 | 1.15 (1.06–1.26) | 1.40 (1.30–1.50) | 1.90 (1.82–1.99) | 2.04 (1.93–2.16) |

**Table S7. Metropolitan and non-metropolitan age-adjusted mortality rates, 1999–2020.**

*AAMR (95% CI) per 100,000 population, by year and urbanization status. CDC WONDER does not provide urbanization classification data beyond 2020.*

| **Year** | **Metropolitan AAMR (95% CI)** | **Non-Metropolitan AAMR (95% CI)** |
| --- | --- | --- |
| 1999 | 1.36 (1.30–1.42) | 1.36 (1.23–1.50) |
| 2000 | 1.25 (1.19–1.30) | 1.25 (1.13–1.37) |
| 2001 | 1.23 (1.17–1.29) | 1.15 (1.04–1.27) |
| 2002 | 1.28 (1.22–1.33) | 1.17 (1.05–1.29) |
| 2003 | 1.19 (1.14–1.25) | 1.28 (1.16–1.41) |
| 2004 | 1.08 (1.03–1.13) | 1.16 (1.05–1.28) |
| 2005 | 1.22 (1.17–1.28) | 1.14 (1.02–1.25) |
| 2006 | 1.14 (1.09–1.19) | 1.19 (1.07–1.31) |
| 2007 | 1.23 (1.18–1.28) | 1.16 (1.05–1.28) |
| 2008 | 1.13 (1.08–1.18) | 1.17 (1.06–1.28) |
| 2009 | 1.15 (1.10–1.20) | 1.06 (0.95–1.17) |
| 2010 | 1.10 (1.06–1.15) | 1.02 (0.92–1.13) |
| 2011 | 1.15 (1.10–1.20) | 1.15 (1.04–1.27) |
| 2012 | 1.20 (1.15–1.25) | 1.22 (1.10–1.34) |
| 2013 | 1.22 (1.17–1.27) | 1.41 (1.28–1.54) |
| 2014 | 1.22 (1.17–1.27) | 1.25 (1.13–1.37) |
| 2015 | 1.26 (1.21–1.30) | 1.49 (1.36–1.62) |
| 2016 | 1.26 (1.21–1.31) | 1.46 (1.33–1.59) |
| 2017 | 1.28 (1.23–1.33) | 1.52 (1.39–1.65) |
| 2018 | 1.27 (1.22–1.32) | 1.65 (1.51–1.79) |
| 2019 | 1.27 (1.22–1.32) | 1.71 (1.58–1.85) |
| 2020 | 1.98 (1.92–2.04) | 2.64 (2.47–2.81) |

**Table S8. Detailed place-of-death category breakdown, 1999–2024.**

*Absolute number of deaths by detailed place-of-death category, prior to grouping into the “Other/Unknown” category shown in Table 1.*

| **Place of Death Category** | **Deaths** | **% of Total** |
| --- | --- | --- |
| Medical Facility - Inpatient | 66833 | 81.74% |
| Medical Facility - Outpatient or ER | 2075 | 2.54% |
| Decedent's Home | 5555 | 6.79% |
| Hospice Facility | 2357 | 2.88% |
| Nursing Home / Long-term Care | 3157 | 3.86% |
| Other | 1472 | 1.80% |
| Medical Facility - Dead on Arrival | 22 | 0.03% |
| Medical Facility - Status Unknown | 68 | 0.08% |
| Place of Death Unknown | 74 | 0.09% |
| Subtotal, named categories | 81613 | 99.82% |
| Suppressed / unclassifiable (implicit remainder) | 146 | 0.18% |
| Total | 81759 | 100.00% |

*Note: “Other/Unknown” in Table 1 (1,782 deaths, 2.18%) combines Other, Dead on Arrival, Status Unknown, Place of Death Unknown, and the suppressed/unclassifiable remainder shown here.*

**Table S9. State-level age-adjusted mortality rates, 1999–2020, ranked by AAMR.**

*States/District of Columbia ranked from highest to lowest AAMR (per 100,000 population).*

| **State** | **Deaths** | **AAMR** | **95% CI** |
| --- | --- | --- | --- |
| New Mexico | 888 | 2.90 | 2.70–3.09 |
| California | 10947 | 2.04 | 2.00–2.08 |
| West Virginia | 633 | 1.93 | 1.78–2.08 |
| Oklahoma | 1068 | 1.83 | 1.72–1.94 |
| Texas | 6241 | 1.81 | 1.76–1.85 |
| Kentucky | 1147 | 1.66 | 1.56–1.76 |
| Rhode Island | 287 | 1.66 | 1.47–1.85 |
| Arizona | 1577 | 1.63 | 1.55–1.71 |
| Tennessee | 1603 | 1.58 | 1.50–1.66 |
| Washington | 1639 | 1.56 | 1.49–1.64 |
| South Carolina | 1103 | 1.47 | 1.39–1.56 |
| South Dakota | 184 | 1.46 | 1.25–1.67 |
| Alaska | 140 | 1.45 | 1.20–1.70 |
| District of Columbia | 125 | 1.45 | 1.19–1.70 |
| Wyoming | 125 | 1.45 | 1.19–1.71 |
| North Dakota | 141 | 1.35 | 1.12–1.57 |
| Montana | 204 | 1.27 | 1.09–1.45 |
| Alabama | 964 | 1.24 | 1.16–1.32 |
| Ohio | 2313 | 1.22 | 1.17–1.28 |
| Indiana | 1235 | 1.21 | 1.14–1.28 |
| Nevada | 492 | 1.21 | 1.10–1.32 |
| Vermont | 130 | 1.21 | 1.00–1.42 |
| North Carolina | 1750 | 1.19 | 1.13–1.24 |
| Colorado | 901 | 1.19 | 1.11–1.27 |
| Arkansas | 557 | 1.18 | 1.08–1.28 |
| Massachusetts | 1249 | 1.17 | 1.11–1.24 |
| Michigan | 1841 | 1.16 | 1.10–1.21 |
| Mississippi | 526 | 1.16 | 1.06–1.26 |
| Delaware | 175 | 1.16 | 0.99–1.34 |
| Oregon | 725 | 1.13 | 1.04–1.21 |
| New Hampshire | 235 | 1.07 | 0.93–1.20 |
| Idaho | 247 | 1.06 | 0.93–1.20 |
| Maryland | 921 | 1.03 | 0.96–1.10 |
| Wisconsin | 918 | 1.02 | 0.95–1.09 |
| Minnesota | 865 | 1.02 | 0.95–1.09 |
| Connecticut | 599 | 1.02 | 0.93–1.10 |
| Florida | 3297 | 1.00 | 0.97–1.04 |
| Hawaii | 226 | 0.98 | 0.85–1.11 |
| Illinois | 1824 | 0.96 | 0.92–1.01 |
| Virginia | 1111 | 0.92 | 0.86–0.97 |
| Louisiana | 621 | 0.92 | 0.85–0.99 |
| Nebraska | 261 | 0.92 | 0.80–1.03 |
| Missouri | 861 | 0.91 | 0.85–0.97 |
| New York | 2839 | 0.90 | 0.86–0.93 |
| New Jersey | 1261 | 0.90 | 0.85–0.95 |
| Maine | 216 | 0.89 | 0.77–1.01 |
| Pennsylvania | 1893 | 0.87 | 0.83–0.91 |
| Utah | 285 | 0.87 | 0.77–0.97 |
| Georgia | 1160 | 0.84 | 0.79–0.89 |
| Kansas | 355 | 0.78 | 0.70–0.86 |
| Iowa | 344 | 0.69 | 0.62–0.77 |

**Table S10. State-level age-adjusted mortality rates, 2021–2024, ranked by AAMR.**

*States/District of Columbia ranked from highest to lowest AAMR (per 100,000 population).*

| **State** | **Deaths** | **AAMR** | **95% CI** |
| --- | --- | --- | --- |
| New Mexico | 349 | 5.62 | 5.02–6.28 |
| Oklahoma | 551 | 4.50 | 4.12–4.91 |
| South Dakota | 104 | 4.09 | 3.30–5.01 |
| Kentucky | 580 | 3.97 | 3.65–4.33 |
| West Virginia | 246 | 3.88 | 3.38–4.45 |
| Arizona | 679 | 2.97 | 2.74–3.21 |
| North Dakota | 67 | 2.94 | 2.26–3.79 |
| Wyoming | 53 | 2.80 | 2.07–3.75 |
| Texas | 2230 | 2.62 | 2.51–2.73 |
| Tennessee | 584 | 2.59 | 2.38–2.82 |
| Colorado | 444 | 2.54 | 2.31–2.80 |
| Montana | 85 | 2.48 | 1.96–3.11 |
| California | 2892 | 2.42 | 2.33–2.52 |
| Indiana | 504 | 2.39 | 2.18–2.61 |
| Alaska | 45 | 2.31 | 1.67–3.16 |
| Nevada | 230 | 2.30 | 2.00–2.62 |
| Mississippi | 207 | 2.28 | 1.97–2.63 |
| Washington | 535 | 2.17 | 1.99–2.37 |
| Nebraska | 113 | 2.07 | 1.69–2.51 |
| Maine | 100 | 2.06 | 1.66–2.56 |
| Arkansas | 195 | 2.00 | 1.72–2.32 |
| South Carolina | 349 | 1.92 | 1.71–2.14 |
| Delaware | 71 | 1.91 | 1.47–2.47 |
| District of Columbia | 33 | 1.88 | 1.29–2.66 |
| North Carolina | 645 | 1.82 | 1.68–1.98 |
| Ohio | 684 | 1.80 | 1.66–1.95 |
| Oregon | 252 | 1.80 | 1.57–2.05 |
| Michigan | 574 | 1.78 | 1.63–1.94 |
| New Hampshire | 85 | 1.78 | 1.40–2.24 |
| Minnesota | 310 | 1.73 | 1.53–1.94 |
| Alabama | 289 | 1.72 | 1.52–1.94 |
| Florida | 1384 | 1.72 | 1.63–1.82 |
| Rhode Island | 60 | 1.58 | 1.19–2.09 |
| Connecticut | 190 | 1.57 | 1.35–1.82 |
| Missouri | 299 | 1.52 | 1.35–1.71 |
| Hawaii | 72 | 1.49 | 1.16–1.90 |
| Maryland | 289 | 1.48 | 1.31–1.67 |
| Wisconsin | 285 | 1.46 | 1.29–1.65 |
| Idaho | 85 | 1.45 | 1.15–1.81 |
| Massachusetts | 336 | 1.45 | 1.29–1.62 |
| Virginia | 394 | 1.40 | 1.26–1.55 |
| Iowa | 139 | 1.39 | 1.16–1.66 |
| Kansas | 128 | 1.39 | 1.15–1.67 |
| Pennsylvania | 624 | 1.37 | 1.26–1.49 |
| Utah | 109 | 1.37 | 1.12–1.66 |
| Vermont | 28 | 1.18 | 0.76–1.79 |
| Georgia | 390 | 1.17 | 1.05–1.30 |
| New Jersey | 352 | 1.14 | 1.02–1.27 |
| Illinois | 453 | 1.13 | 1.02–1.24 |
| Louisiana | 149 | 1.03 | 0.86–1.22 |
| New York | 659 | 1.01 | 0.93–1.09 |

**Table S11. COVID-19 (U07.1) and case-definition sensitivity analyses.**

*Results of sensitivity analyses assessing whether observed trends were attributable to COVID-19-specific coding or the narrower primary case definition.*

**S11a. Deaths with COVID-19 (U07.1) as the underlying cause among primary case-definition deaths, 2020–2024.**

| **Year** | **Total co-mention deaths** | **U07.1-underlying deaths (%)** | **AAMR, U07.1-underlying subset** |
| --- | --- | --- | --- |
| 2020 | 5383 | 1,382 (25.67%) | 0.51 |
| 2021 | 6085 | 2,096 (34.45%) | 0.77 |
| 2022 | 5478 | 1,292 (23.59%) | 0.47 |
| 2023 | 4331 | 268 (6.19%) | 0.11 |
| 2024 | 4616 | 184 (3.99%) | 0.06 |

*2019: no deaths met the U07.1-underlying definition. 2020–2024 total: 25,893 primary-definition deaths; 5,222 (20.17%) with U07.1 as underlying cause.*

**S11b. Joinpoint trend analysis restricted to 1999–2019 (pre-pandemic period only).**

| **Analysis** | **Year Interval** | **APC (95% CI)** | **p-Value** |
| --- | --- | --- | --- |
| Pre-pandemic (1999–2019 data only) | 1999–2009 | -1.36* (-2.22 to -0.49) | 0.005 |
| Pre-pandemic (1999–2019 data only) | 2009–2019 | 1.98* (1.26 to 2.71) | <0.001 |
| AAPC, full range | 1999–2019 | 0.30 (-0.22 to 0.82) | 0.263 |

*This analysis differs from S11c below: here, the Joinpoint model was run using only 1999–2019 data (2020–2024 not included), rather than modeling the full 1999–2024 series with 2020–2022 omitted as data points.*

**S11c. Joinpoint trend analysis excluding 2020–2022 (pandemic period) from the full 1999–2024 series.**

| **Analysis** | **Year Interval** | **APC (95% CI)** | **p-Value** |
| --- | --- | --- | --- |
| Overall (2020–2022 omitted) | 1999–2010 | -1.33* (-2.21 to -0.43) | 0.006 |
|  | 2010–2024 | 2.78* (2.16 to 3.39) | <0.001 |
| AAPC, full range | 1999–2024 | 0.95* (0.46 to 1.44) | <0.001 |

**S11d. Joinpoint trend analysis using alternative diagnostic code definitions.**

| **Case Definition** | **Year Interval** | **APC (95% CI)** | **p-Value** |
| --- | --- | --- | --- |
| CLD restricted to K74 only | 1999–2009 | -3.28* (-4.47 to -2.08) | <0.001 |
|  | 2009–2018 | 1.35 (-0.64 to 3.38) | 0.169 |
|  | 2018–2021 | 22.51* (6.45 to 40.98) | 0.008 |
|  | 2021–2024 | -12.50* (-17.43 to -7.28) | <0.001 |
| Pneumonia broadened to J09–J18 | 1999–2009 | -1.35* (-2.58 to -0.10) | 0.036 |
|  | 2009–2018 | 2.13* (0.68 to 3.59) | 0.007 |
|  | 2018–2021 | 19.37* (6.31 to 34.04) | 0.005 |
|  | 2021–2024 | -9.87* (-15.25 to -4.13) | 0.003 |
| CLD broadened to K70–K76 + B18 | 1999–2018 | -0.81* (-1.30 to -0.31) | 0.003 |
|  | 2018–2021 | 25.82* (7.63 to 47.08) | 0.006 |
|  | 2021–2024 | -14.31* (-19.33 to -8.97) | <0.001 |

** Indicates APC significantly different from zero at α = 0.05. All alternative case definitions retained the same overall pattern (rise 2018–2021, decline 2021–2024) observed in the primary analysis.*
